# Temperature-specific regulation of the NDR kinase Orb6 by the MAPK Sty1 to promote heat stress resilience

**DOI:** 10.1242/jcs.264507

**Published:** 2026-04-22

**Authors:** Laura P. Doyle, Robert N. Tams, Chuan Chen, Illyce Nuñez, Patrick Roman Haller, Fulvia Verde

**Affiliations:** Department of Molecular and Cellular Pharmacology, University of Miami, Miami, FL, USA

**Keywords:** NDR kinase, Orb6, Mitogen-activated protein kinase, Sty1, Cdc42 GTPase, Sts5, RNP granule assembly, Heat stress, *Schizosaccharomyces pombe*

## Abstract

The cellular response to environmental fluctuations, such as increased temperature, is crucial in promoting cell survival and plays an increasingly recognized role in cancer biology. Important cellular functions altered by heat stress are cell polarization and protein translation. Previous studies have shown that heat stress alters the dynamics of Cdc42, a key regulator of cell polarization in eukaryotes, and promotes ribonucleoprotein (RNP) granule formation, reprogramming protein translation. The biological mechanisms underlying these vast changes are only partially known. Here, we report that the conserved NDR kinase Orb6, a homolog of mammalian STK38, responds to heat stress and regulates heat stress resilience by modulating Cdc42 dynamics and promoting RNP granule assembly in *Schizosaccharomyces pombe*. Also, we discovered a finely tuned mechanism whereby stress-activated mitogen-activated protein kinase (MAPK) Sty1 negatively regulates Orb6 kinase and Orb6 C-terminal phosphorylation during heat stress. Orb6 inhibition by Sty1 increases the sensitivity of the cell to heat stress in a temperature-specific manner, fostering increased stress resilience and metabolic adaptation. These observations highlight the role of NDR kinase in the process of heat adaptation and thermotolerance during environmental cell exposure to elevated temperatures.

## Teaser

**Summary:** The inhibition of the nuclear Dbf2-related kinase Orb6 by stress-activated protein kinase Sty1 promotes heat stress resilience in a temperature-specific manner.

## INTRODUCTION

The ability to respond to varying environmental conditions, such as elevated temperature, is a vital cellular defense mechanism that promotes adaptation and survival (Zayani et al., 2025). The heat stress response activates adaptive mechanisms including antioxidant defenses, metabolic shifts and induction of heat-shock proteins (HSPs) (Jung et al., 2013; Gomez-Pastor et al., 2018; Ciocca et al., 2013; Hu et al., 2022; Ding and Gao, 2025). Activation of the heat-shock response usually provides cytoprotective effects, and HSPs have been found to promote survival of cancer cells (Wu et al., 2017). In eukaryotes, severe heat stress (typically ≥42°C) rapidly disrupts cellular function, triggering a robust heat shock response, protein denaturation and aggregation, as well as a decrease in protein synthesis.

During heat stress, cells undergo distinct morphological changes. Mammalian cells often lose their spread morphology and become more rounded due to cytoskeletal reorganization (Velichko et al., 2013; Dressler et al., 2005; Gungor et al., 2014). However, the mechanisms that regulate morphological changes during heat stress are poorly understood. In the fission yeast *Schizosaccharomyces pombe*, an amenable model system, mild, sublethal heat stress at 36°C alters cell polarization and the dynamic behavior of Cdc42 GTPase (Vjestica et al., 2013), a key regulator of cell morphology in eukaryotes (Schwamborn and Püschel, 2004; Stankiewicz and Linseman, 2014). In unstressed conditions, Cdc42 displays oscillatory dynamics at cell tips (Das et al., 2012). Conversely, during heat stress (Vjestica et al., 2013), as well as in response to specific environmental conditions, such as pheromone exposure (Bendezú and Martin, 2013), oxidative stress (Salat-Canela et al., 2021) or nitrogen starvation (Chen et al., 2019), an alternative pattern of Cdc42 emerges, where dynamic patches of active Cdc42 appear and disappear throughout the cell membrane. Although the emergence of ectopic Cdc42 dynamics involves the activation of the conserved stress-activated mitogen-activated protein kinase (MAPK) Sty1 (also known as Spc1) (Salat-Canela et al., 2021; Mutavchiev et al., 2016; Doyle et al., 2025), the mechanism that regulates Cdc42 dynamics shift during thermal stress is poorly understood.

During heat stress, cells also rapidly suppress global protein synthesis to conserve energy and prevent accumulation of misfolded protein by the inhibition of translation initiation and mRNA sequestration into stress granules (SGs) and P-bodies for storage or decay (Kedersha et al., 2005; Grousl et al., 2009). SGs are membraneless cytoplasmic aggregates that form in response to various stress conditions, including heat shock, oxidative stress, osmotic stress and infections (Marcelo et al., 2021). Their primary role is to protect mRNA and regulate translation during stress, helping cells survive adverse conditions. The formation process relies on liquid–liquid phase separation (LLPS), driven by multivalent interactions between RNA-binding proteins (RBPs) and untranslated mRNAs. SGs are crucial adaptive structures that integrate translational control with cellular stress signaling (Desai et al., 2026). Dysregulation of SG dynamics is increasingly recognized as a contributor to human disease and is strongly linked to neurodegeneration (ALS and FTD), cancer and viral infections (Ramaswami et al., 2013; Anderson et al., 2015; Aguzzi and Altmeyer, 2016; Gilks et al., 2004; Alberti and Dormann, 2019).

Nuclear Dbf2-related (NDR) kinases are highly conserved members of the AGC protein kinase family that regulate essential cellular processes including morphogenesis, growth and proliferation, mitosis and apoptosis (Hergovich et al., 2006; Cornils et al., 2011; Yang et al., 2014a; Vichalkovski et al., 2008; Verde et al., 1995; Chiba et al., 2009; Hergovich et al., 2007, 2009; Verde et al., 1998). Several studies have implicated dysregulation of NDR kinases in the development of cancer (Cornils et al., 2010; Zhang et al., 2015; Adeyinka et al., 2002; Millward et al., 1998). NDR kinases also play a role in neuronal differentiation (Emoto et al., 2004; Yang et al., 2014; Zallen et al., 2000; Demiray et al., 2018). The fission yeast *S. pombe* has an NDR/LATS kinase known as Orb6, which is responsible for regulation of polarized cell growth (Verde et al., 1995). We have previously reported that Orb6 kinase has separate roles in the control of Cdc42 GTPase (Das et al., 2015, 2009) and mRNA translation (Nuñez et al., 2016; Chen et al., 2019). Orb6 governs cell polarity by promoting the Ras1-dependent Cdc42 control axis (Chen et al., 2019) and by inhibiting a stress-activated Cdc42 module (Doyle et al., 2025; Salat-Canela et al., 2021) composed of the guanine nucleotide exchange factor (GEF) Gef1 (Das et al., 2015, 2012, 2009; Coll et al., 2003) and the Cdc42 GTPase-activating protein (GAP) Rga3 (Doyle et al., 2025; Gallo Castro and Martin, 2018). Downregulation of Orb6 kinase, which suppresses Ras1 GTPase and derepresses the stress-activated Cdc42 module, promotes ectopic Cdc42 activation (Chen et al., 2019; Das et al., 2009).

Orb6 kinase also fosters polarized cell growth by spatiotemporal regulation of the RBP Sts5 (Nuñez et al., 2016; Chen et al., 2019). Sts5 is homologous to human Dis3L2, which is associated with Perlman's syndrome and Wilm's tumor in humans (Astuti et al., 2012; Lv et al., 2015; Robinson et al., 2015; Malecki et al., 2013; Toda et al., 1996; Vaggi et al., 2012; Jansen et al., 2009; Kurischko et al., 2011). Sts5 functions to repress translation of specific mRNAs, many of which encode proteins involved in polarized cell growth, via binding and sequestration into ribonucleoprotein (RNP) granules (Nuñez et al., 2016), under conditions of nutritional stress (Nuñez et al., 2016; Chen et al., 2019; Magliozzi and Moseley, 2021). Environmental stress, such as nitrogen starvation, or crowded growth conditions leading to stationary phase, inhibit Orb6 kinase activity, which results in the translational repression of mRNAs involved in polarized growth via formation of Sts5 granules (Nuñez et al., 2016; Chen et al., 2019).

In this paper, we report that heat stress downregulates Orb6 kinase activity, promoting ectopic Cdc42 activation and Sts5 coalescence into granules. Furthermore, we find that Orb6 kinase inhibition promotes cell resilience to heat stress. Recently, we have reported that stress activated MAPK Sty1 negatively regulates Orb6 kinase activity during nitrogen starvation (Doyle et al., 2025). Here, we show that the MAPK Sty1 also has a role in the induction of ectopic Cdc42 activation, Sts5 granule assembly and the inhibition of Orb6 kinase activity during mild heat stress. Furthermore, we find that the levels of phosphorylation of Orb6-Thr456, in the C-terminal hydrophobic motif, are decreased upon heat stress and that Sty1 activation enhances the sensitivity of Thr456 phosphorylation and Orb6 kinase function to heat stress. Thus, our observations identify a temperature-specific regulation of the NDR kinase Orb6 by the MAPK Sty1 to promote ectopic Cdc42 activation and stress resilience.

## RESULTS

### Orb6 kinase function and activity are downregulated by heat stress

**Fig. 1.**
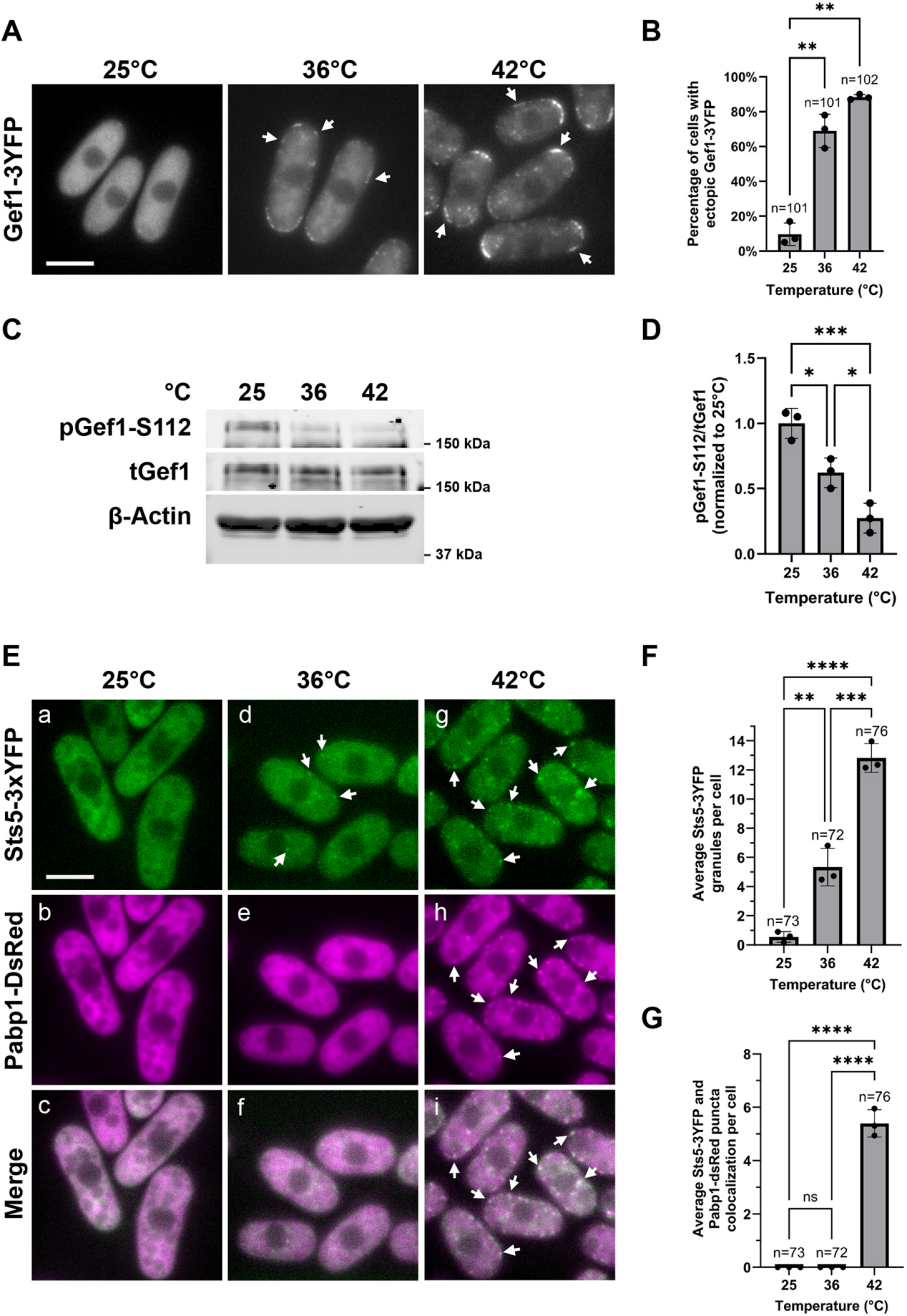
**Orb6 activity is negatively regulated by heat stress.** (A) Gef1–3YFP localizes to the cell membrane upon heat exposure at 36°C or 42°C for 30 min (arrows). (B) Quantification of the percentage of cells that display ectopic Gef1–3YFP localization from experiments as in A based on three independent experiments. Data presented as mean±s.d. *n*, number of cells quantified across all three independent experiments. **P≤0.01 (one-way ANOVA with Tukey's HSD test). (C) Orb6 activity, as measured by Gef1-S112 phosphorylation, decreases after 30 min at 36°C or 42°C. (D) Quantification of pGef1-S112/total (t)Gef1 from experiments as in C upon temperature stress exposure based on three independent experiments. Data are presented as in B. *P≤0.05; ***P≤0.001. (E) *Sts5-3YFP pabp1-DsRed* cells were exposed to 36°C or 42°C for 30 min. At 36°C, Sts5–3YFP formed cytoplasmic puncta (d, arrows) but no SG formation was visible (f). At 42°C, Sts5 partially colocalized with the SG marker Pabp1–DsRed (g–i, arrows). (F) Quantification of average number of Sts5 puncta present in each cell from E based on three independent experiments. *n*=number of cells quantified across all three independent experiments. Data are presented as in B. **P≤0.01; ***P≤0.001; ****P≤0.0001. (G) Quantification of average number of Sts5 puncta that colocalized with the SG marker Pabp1 in each cell from experiments as in E based on three independent experiments. Data are presented as in B. *n*=number of cells quantified across all three independent experiments. ****P≤0.0001; ns, not significant. Scale bars: 5 μm.

We have previously shown that Orb6 kinase is sensitive to nutritional stress, and that downregulation of Orb6 kinase activity alters cell morphology and protein translation during nitrogen deprivation (Chen et al., 2019). During nutritional stress, Orb6 kinase activity decreases, and therefore phosphorylation of the Cdc42 control factors and Orb6 substrates Gef1 (a Cdc42 GEF) and Rga3 (a Cdc42 GAP) declines, promoting ectopic activation of Cdc42 (Das et al., 2015; Chen et al., 2019; Doyle et al., 2025). The onset of ectopic Cdc42 activation and increased Gef1 membrane localization were previously reported to also occur during exposure to sublethal mild heat stress at 36°C (Vjestica et al., 2013). Therefore, we investigated whether Orb6 kinase has a role in the cellular response to heat stress. Consistent with previous findings, we found that Gef1–3YFP localized to the cell membrane after 30 min at 36°C (Fig. 1A,B) as compared to the control, where Gef1 remained diffusely localized in the cytoplasm at 25°C. Furthermore, we found that Gef1–3YFP also localized to the cell membrane at the higher severe heat stress temperature of 42°C (Fig. 1A,B).

As Gef1 is a substrate that is phosphorylated by Orb6 at serine 112, we sought to measure Orb6 activity using a previously described custom phospho-specific antibody for Gef1-S112 (Chen et al., 2019). We found that Gef1-S112 phosphorylation decreased after 30 min at 36°C, a sublethal temperature that induces heat stress, which was further exacerbated at 42°C, a lethal temperature that induces a severe heat-shock response (Fig. 1C,D). These results indicate the Orb6 activity decreases during both mild and severe heat stress exposure, fostering ectopic activation of Cdc42.

During heat exposure, cells reorganize their RNA and protein metabolism by forming two major types of cytoplasmic granules: SGs (Guzikowski et al., 2019; Mahboubi and Stochaj, 2017) and processing bodies (P-bodies). Both are membraneless organelles formed by LLPS, but they have distinct roles and compositions. We have previously demonstrated that Orb6 kinase controls polarized cell growth and protein translation by phosphorylating the highly conserved RBP Sts5. Sts5, an Orb6 substrate, remains diffused in the cytoplasm when Orb6 is active, whereas it partially colocalizes with P-bodies following Orb6 inhibition, or in media lacking glucose or nitrogen (Nuñez et al., 2016; Magliozzi and Moseley, 2021).

Consistent with Orb6 kinase activity being downregulated by heat exposure, we found that Sts5–3YFP remained diffused in the cytoplasm at 25°C, whereas it formed puncta at both 36°C and 42°C (Fig. 1E,F). We found that Sts5–3YFP puncta readily associated with SGs (using Pabp1–dsRed SG marker) at 42°C (Fig. 1Eg–i,G), but not at 36°C where we find no SG assembly (Fig. 1Ed–f,G). Conversely, we find that Sts5–3YFP colocalizes with P-body marker Dcp1–mCherry at both 36°C and 42°C (Fig. S1).

We confirmed Sts5 and SG colocalization, under conditions of glucose limitation (Fig. S2A), which strongly induces SG formation (Nilsson and Sunnerhagen, 2011), as well as Sts5 aggregation into RNP granules (Nuñez et al., 2016). We also found that *ssp1* mRNA, a Sts5-bound mRNA that is translationally repressed upon formation of Sts5 puncta (Nuñez et al., 2016), was colocalized with SG marker Pabp1–dsRed by RNA-FISH analysis (Fig. S2A). *ssp1* encodes a CamK kinase (Hanyu et al., 2009; Matsusaka et al., 1995). We have previously used *ssp1* mRNA to detect colocalization of Sts5 with P-bodies (Nuñez et al., 2016).

**Fig. 2.**
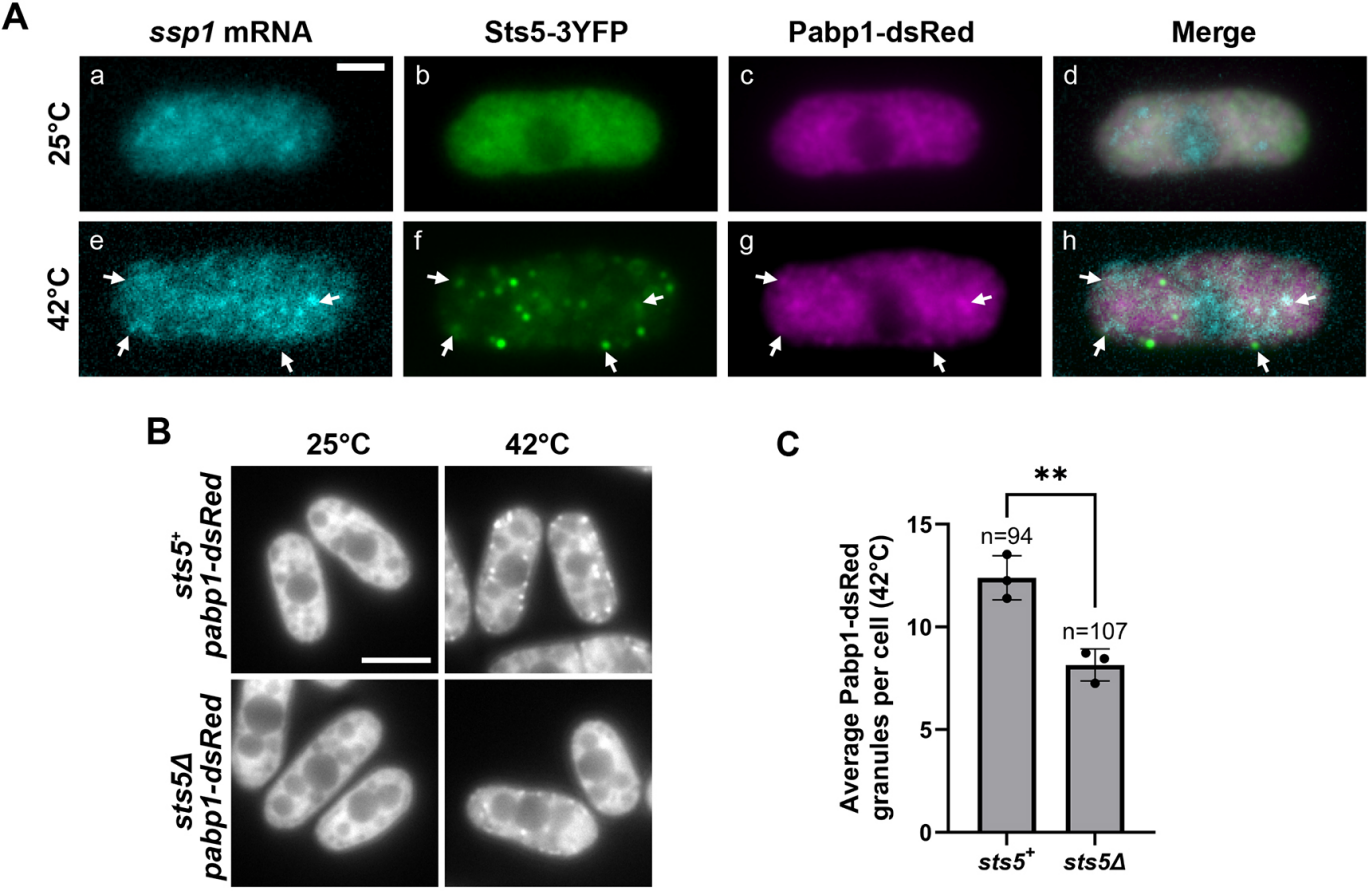
**Orb6 and Sts5 regulate SG formation.** (A) RNA FISH visualization of *ssp1* mRNA in fixed cells cultured for 30 min at 42°C. Hybridization of RNA was performed with 20-mer DNA oligonucleotides (Stellaris) labeled with Quasar 705 fluorochromes. *ssp1* mRNA colocalized with Sts5–3YFP and the SG marker Pabp1–DsRed (arrows) upon heat stress at 42°C but not at 25°C control conditions. (B) *pabp1-DsRed* and *sts5Δ pabp1-DsRed* cells were exposed to 42°C for 30 min or kept at 25°C. At 25°C, the SG marker Pabp1–DsRed remained diffuse throughout the cytoplasm. Following heat stress at 42°C, *sts5Δ* cells formed less SGs than the control. (C) Quantification of average number of SG marker Pabp1-dsRed puncta assembly at 42°C from B based on three independent experiments. Data presented as mean±s.d., *n*=number of cells quantified across all three independent experiments. **P≤0.01 (two-tailed unpaired *t*-test). Scale bars: 5 μm.

During heat stress, similar to what was observed in glucose deprivation conditions, we observed colocalization of Sts5 puncta, *ssp1* mRNA and SGs in cells exposed to 42°C for 30 min, as compared to 25°C controls (Fig. 2A). Overall, these findings show that Orb6 kinase activity is downregulated upon heat stress and heat shock, fostering ectopic activation of Cdc42 and coalescence of the RBP Sts5 into SGs and P-bodies.

### Sts5 aggregation promotes SG assembly

We have previously demonstrated that Orb6 inhibition promotes the formation of P-bodies in a Sts5-dependent manner (Nuñez et al., 2016), suggesting that Sts5 aggregates might also promote RNP granule formation during heat stress. To test whether Sts5 plays a role in SG nucleation, we followed the formation of SGs in the *sts5Δ* mutant using the SG marker Pabp1–DsRed. In this experiment, we induced SGs via heat stress at 42°C, which induces SG assembly. We found that the number of SGs decreased by 35% in *sts5Δ* mutant cells as compared to control cells (Fig. 2B,C) after exposure to 42°C for 30 min. Cells were also exposed to glucose deprivation and SG formation decreased 44% in *sts5Δ* mutants (Fig. S2B,C). At the control temperature of 25°C, Pabp1–DsRed is largely diffuse within the cytoplasm in both the *sts5Δ* and control strains (Fig. 2B). Overall, these data indicate that the presence of Sts5 is important for the formation of SGs.

### Sts5 aggregation correlates with heat resilience in *S. pombe*

**Fig. 3.**
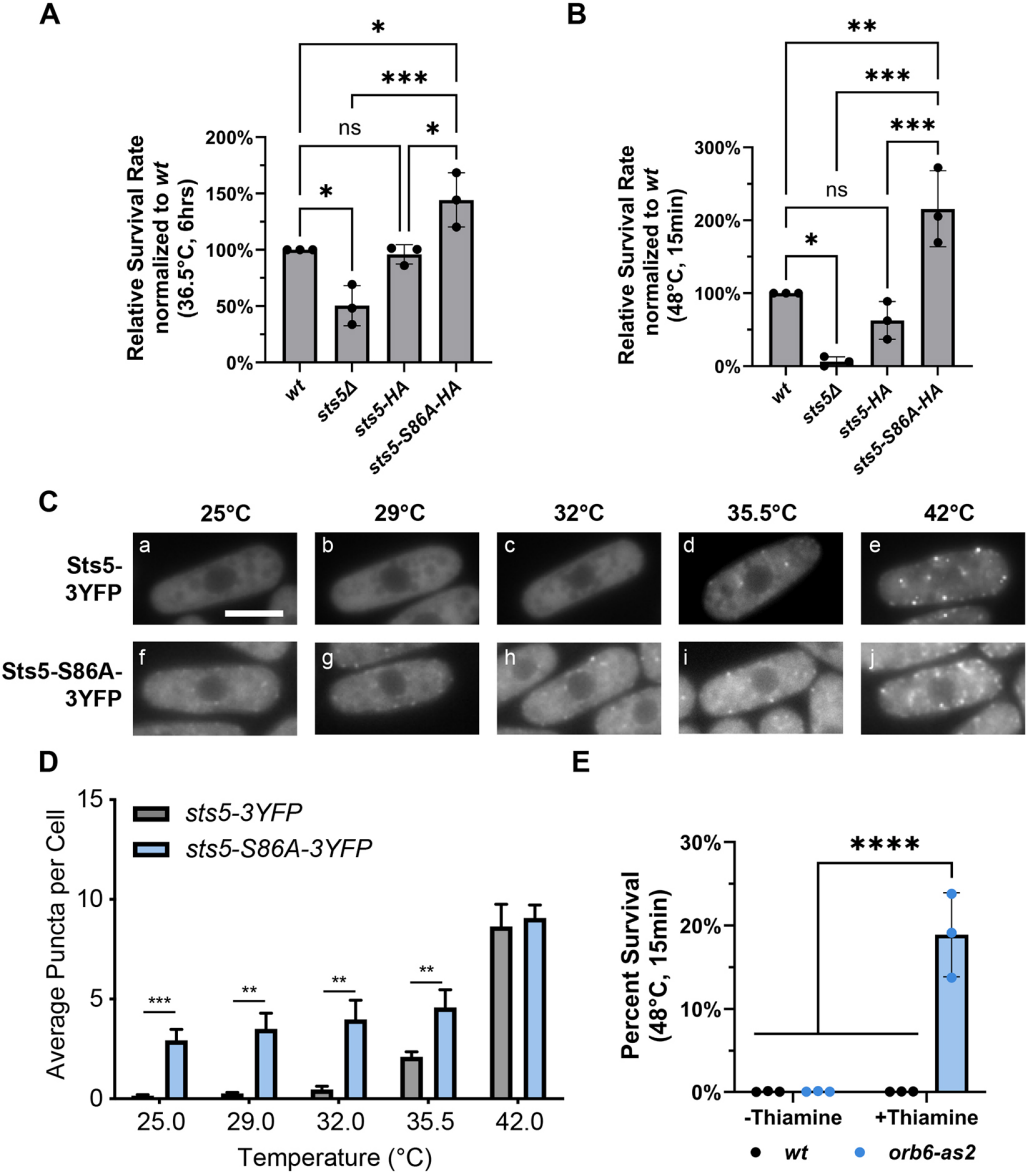
**Orb6 and Sts5 modulate survival following heat stress.** (A) Wild-type (*wt*), *sts5Δ*, *sts5-HA* and *sts5-S86A-HA* cells were grown for 6 h at either 25°C or 36.5°C. Following prolonged heat stress at 36.5°C, s*ts5Δ* cells displayed decreased survival compared to wild-type, whereas *sts5-S86A-HA* showed increased survival compared to the *sts5-HA* control. (B) The experiment in A was repeated, but cells were instead incubated for 15 min at 48°C temperature, whereas untreated controls were incubated at 25°C. Again, *sts5Δ* cells exhibit decreased survival, whereas the *sts5-S86A-HA* mutant exhibited increased survival. Data in A and B are presented as mean±s.d., *n*=3. *P≤0.05; **P≤0.01; ***P≤0.001; ns, significant (one-way ANOVA with Tukey's HSD test). (C) In the *sts5-S86A-3YFP* mutant, the number of Sts5 granules were increased at temperatures in which the *sts5-3YFP* control did not form granules. Scale bar: 5 μm. (D) Quantification from results obtained from experiments as in C. There is a statistically significant increase in average puncta per cell for the *sts5-S86A-3YFP* strain as compared to the control for cells incubated between 25°C and 35.5°C. Data are presented as mean±s.d., *n*=60 cells quantified across all three independent experiments. **P≤0.01; ***P≤0.001 (two-way ANOVA with Tukey's HSD test). (E) Wild-type (*wt*) and *orb6-as2* strains were cultured in the presence (+thiamine) or absence of thiamine (−thiamine), heat shocked and plated. Inhibition of Orb6 results in a 17-fold increase in survival as compared to the untreated control. Data are presented as mean±s.d., *n*=3. ****P≤0.0001 (two-way ANOVA with Tukey's HSD test).

SG formation provides a mechanism to protect cells from environmental stress and promote cell survival (Mahboubi and Stochaj, 2017). These condensates can modulate susceptibility to heat stress (Riback et al., 2017; Kroschwald and Alberti, 2017). As Sts5 responds to heat by colocalizing with both SGs and P-bodies (Fig. 1E; Fig. S1) and is important for SG formation (Fig. 2B), we hypothesized that modulating the assembly of Sts5 into RNP granules plays a role in cell protection from heat stress. Sts5 contains an intrinsically disordered domain (IDD), which is phosphorylated by Orb6 kinase at serine 86 (Chen et al., 2019). Modification of Sts5 serine 86 to alanine promotes increased Sts5 puncta formation, increases cell resilience during nutritional stress and promotes chronological lifespan extension (Chen et al., 2019). Thus, we tested whether loss of Sts5 (*sts5Δ*), or the presence of the *sts5-S86A* mutation affected cell survival during extended heat stress or heat shock. To test the role of Sts5 during heat stress and in heat tolerance (exposure to heat for extended times), wild-type, *sts5Δ*, *sts5-HA* and *sts5-S86A-HA* log-phase cells were incubated for 6 h at the heat stress temperature (36.5°C) or control temperature (25°C) in a shaking incubator and plated to calculate the number of colony-forming units (CFUs) (see Materials and Methods). In these assays, we define survival as the ability for the cell to proliferate and form a colony. We found that the *sts5Δ* strain exhibited decreased survival compared to that of the wild type (Fig. 3A). Conversely, the *sts5-S86A-HA* strain displayed increased survival, as compared to the *sts5-HA* or wild-type controls (Fig. 3A). The *sts5-HA* strain exhibited similar survival as compared to the wild type, as expected. We have previously established that addition of the *sts5-S86A* mutation does not alter Sts5 protein levels (Chen et al., 2019).

To test response upon acute heat shock, wild-type, *sts5Δ*, *sts5-HA* and *sts5-S86A-HA* strains were exposed to a 48°C heat shock for 15 min. We found that the *sts5Δ* strain exhibited decreased survival as compared to the wild-type control (Fig. 3B), whereas the *sts5-S86A-HA* mutant exhibited increased survival as compared to either the wild-type or *sts5-HA* controls (Fig. 3B). Similar results were found using *sts5-3YFP* and *sts5-S86A-3YFP* strains (Fig. S3).

Thus, our data suggest that Sts5 coalescence has implications in promoting cell survival and heat stress resilience following various durations and intensities of exposure to elevated temperatures. Therefore, we tested whether Sts5 granule assembly is modulated by increased temperature and whether the *sts5-S86A* mutation increases formation of Sts5 puncta following exposure to heat. To do this, *sts5-3YFP* and *sts5-S86A-3YFP* strains were cultured at 29°C, 32°C, 35.5°C or 42°C for 30 min, whereas control *sts5-3YFP* and *sts5-S86A-3YFP* cells were maintained at 25°C throughout the experiment. Following heat stress, fluorescence microscopy was performed and the number of Sts5 puncta were counted at each temperature. The *sts5-3YFP* control began to form visible puncta at 35.5°C (Fig. 3Ca–d), whereas the *sts5-S86A-3YFP* mutant had already begun to form puncta at 25°C (Fig. 3Cf–j). These differences were statistically significant (Fig. 3D), and the predisposition of *sts5-S86A* cells to form of puncta was readily apparent until the temperature reached 42°C at which point the control formed approximately the same number of puncta (Fig. 3Ce,j,D).

Our findings indicate an important role for serine 86 phosphorylation in modulating the extent of Sts5 puncta assembly in response to increased temperatures (Fig. 3C,D). As Orb6 kinase phosphorylates serine 86 (Chen et al., 2019), we tested whether downregulation of Orb6 kinase also increases cell resilience to heat stress. To transcriptionally repress Orb6, a thiamine-repressible o*rb6-as2* strain was used (see Materials and Methods). Log phase wild-type and *orb6-as2* cells were grown with or without 15 µM thiamine for 16 h at 32°C. Samples were diluted to an equivalent optical density and were heat shocked at 48°C for 15 min whereas control samples were maintained at 32°C. The *orb6-as2* strain, in the presence of thiamine, exhibited a striking increase in survival following heat shock compared to the untreated control (Fig. 3E). This striking increase in resilience was readily observed on Petri plates (Fig. S4) and was notably higher than the relative increase in heat resilience observed for the *sts5-S86A* mutant (Fig. 3A,B). Overall, these data indicate that the loss of Orb6 kinase modulates RNP granule assembly and promotes resilience to heat stress.

### Conserved MAPK Sty1 negatively regulates Orb6 activity in a temperature specific manner

**Fig. 4.**
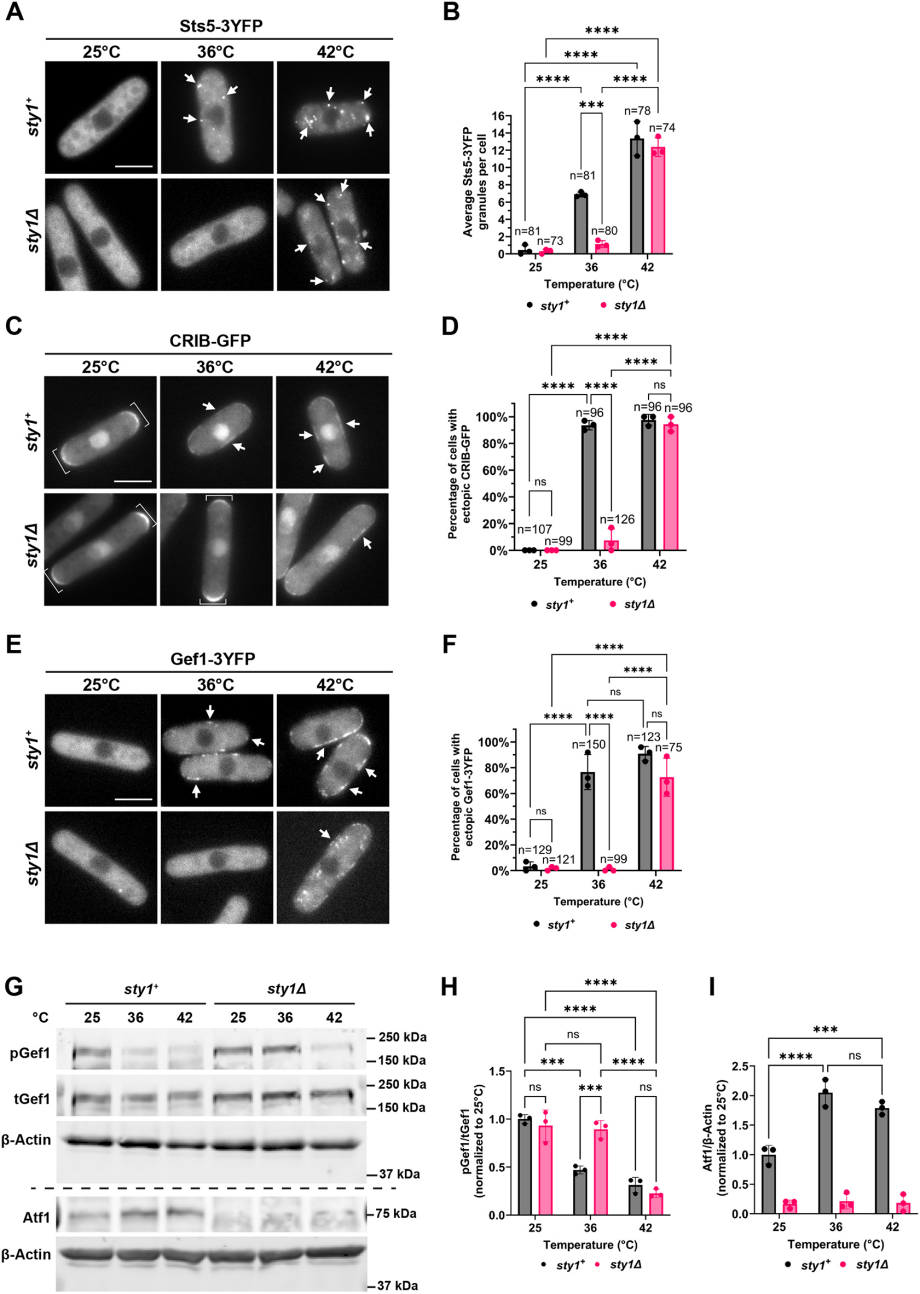
**The distribution of Sts5, Gef1 and active Cdc42 is temperature sensitive and is modulated by Sty1 in a temperature-specific manner.** (A) Sts5–3YFP forms puncta after 30 min at 36°C and 42°C (arrows). In the absence of *sty1,* Sts5–3YFP only clusters at 42°C. Images are a maximum projection *Z*-stack of six images separated by a step-size of 0.3 μm. (B) Quantification of average number of Sts5 puncta present in each cell from A based on three independent experiments. Data are presented as mean±s.d., *n*=number of cells quantified across all three independent experiments. ***P≤0.001; ****P≤0.0001 (two-way ANOVA with Tukey's HSD test). (C) Active Cdc42 forms ectopic patches upon exposure to 36°C and 42°C when *sty1* is present but only forms patches at 42°C when *sty1* is absent (arrows). Active Cdc42 is localized at the cell tips in control cells at 25°C and in *sty1Δ* cells at 25°C and 36°C (brackets). Images are a maximum projection *Z*-stack of six images separated by a step-size of 0.3 μm. (D) Quantification of percentage of cells that display ectopic CRIB–GFP localization from experiments as in C based on three independent experiments. Data presented as in B, *n*=number of cells quantified across all three independent experiments. ****P≤0.0001. (E) Gef1–3YFP localizes to the cell membrane at 36°C and 42°C (arrows). Loss of *sty1* leads to Gef1-3YFP localization to the cell membrane only at 42°C. (F) Quantification of percentage of cells that display ectopic Gef1-3YFP localization from B based on three independent experiments. Data presented as in B. *n*=number of cells quantified across all three independent experiments. ****P≤0.0001; ns, not significant. (G) Gef1-S112 phosphorylation by Orb6 decreases after 30 min at 36°C or 42°C. In *sty1Δ* deletion cells, pGef1-S112 remains constant at 36°C but decreases at 42°C. Atf1 levels increase upon exposure to 36°C or 42°C for 30 min in control cells. β-actin was used as a loading control. (H) Quantification of pGef1-S112/total (t)Gef1 from experiments as in G upon temperature stress exposure in control or *sty1Δ* deletion mutant cells based on three independent experiments. Data are presented as mean±s.d. ***P≤0.001; ****P≤0.0001 (two-way ANOVA with Tukey's HSD test). (I) Quantification of Atf1/actin from experiments as in G upon temperature stress in control or *sty1Δ* deletion mutant cells based on three independent experiments. Data are presented as mean±s.d. ***P≤0.001; ****P≤0.0001 (two-way ANOVA with Tukey's HSD test). Scale bars: 5 μm.

The MAPK Sty1 is activated upon exposure to diverse stressors such as nitrogen starvation, osmotic changes, oxidative stress and heat shock (Toone and Jones, 1998; Shiozaki and Russell, 1996; Millar et al., 1995; Degols et al., 1996). Recently, we have shown that Sty1 activation during nitrogen starvation negatively regulates Orb6 activity (Doyle et al., 2025). Therefore, we tested whether Sty1 has a role in Orb6 regulation during heat stress, by visualizing Sts5–3YFP aggregation during either mild or severe heat stress (36°C or 42°C) for 30 min in the presence or absence of *sty1.* We found that upon loss of *sty1*, Sts5–3YFP did not assemble into granules at 36°C but it still assembled into granules at 42°C (Fig. 4A). The number of Sts5 granules per cell at 42°C is similar in both the presence or absence of *sty1* (Fig. 4B). These results indicate that Sts5 granule assembly during heat stress is dependent on the presence of Sty1 at 36°C but not at 42°C.

Previously, we have shown that Orb6 phosphorylates two regulators of Cdc42, the GEF Gef1 (Das et al., 2015) and the GAP Rga3 (Doyle et al., 2025), to spatially regulate Cdc42 dynamics. When Orb6 is inhibited during nitrogen starvation, Gef1 and Rga3 move to the cell membrane and facilitate the onset of ectopic Cdc42 activation, where active Cdc42 forms dynamic patches along the cell membrane (Doyle et al., 2025). These alternative Cdc42 dynamics are also found upon different stress exposure, including heat stress at 36°C (Vjestica et al., 2013). Thus, we sought to investigate whether ectopic activation of Cdc42 is dependent on Sty1 upon heat exposure. We visualized active Cdc42 using fluorescently tagged Cdc42-Rac interactive binding domain bioreporter (CRIB–GFP) or the Cdc42 GEF Gef1 using Gef1–3YFP. We found that ectopic activation of Cdc42 and Gef1–3YFP membrane localization were induced by heat exposure at both 36°C and 42°C and that these effects were dependent on Sty1 at 36°C, but not at 42°C (Fig. 4C–F).

Furthermore, we tested the effects of heat stress and heat shock on Gef1-S112 phosphorylation by Orb6 kinase, in the presence and absence of *sty1.* We found that Gef1-S112 phosphorylation decreased during exposure to 36°C and 42°C for 30 min (Fig. 4G,H). In the absence of *sty1*, we found that Gef1-S112 phosphorylation remained constant at 36°C but decreased to similar levels to that in wild-type at 42°C (Fig. 4G,H). After 60 min, pGef1-S112 recovered to basal levels in the control strain but remained unchanged in the *sty1* deletion mutant (Fig. S5). Sty1 activation was validated by measuring levels of Atf1, a transcription factor phosphorylated by Sty1 upon Sty1 activation (Shiozaki et al., 1998; Wilkinson et al., 1996; Sun et al., 2024). As expected, we found that Atf1 levels increase upon cell exposure to 36°C or 42°C for 30 min (Fig. 4G,I), confirming that Sty1 is activated under these experimental conditions.

Overall, these results indicate that Sty1 kinase activation during temperature stress exerts a temperature-specific effect on Orb6 kinase activity; Sty1 activation is necessary to inhibit Orb6 activity and biological functions at 36°C whereas is not necessary at higher, heat shock temperatures (42°C).

### Phosphorylation of Orb6-T456 in the C-terminal hydrophobic motif is temperature sensitive

**Fig. 5.**
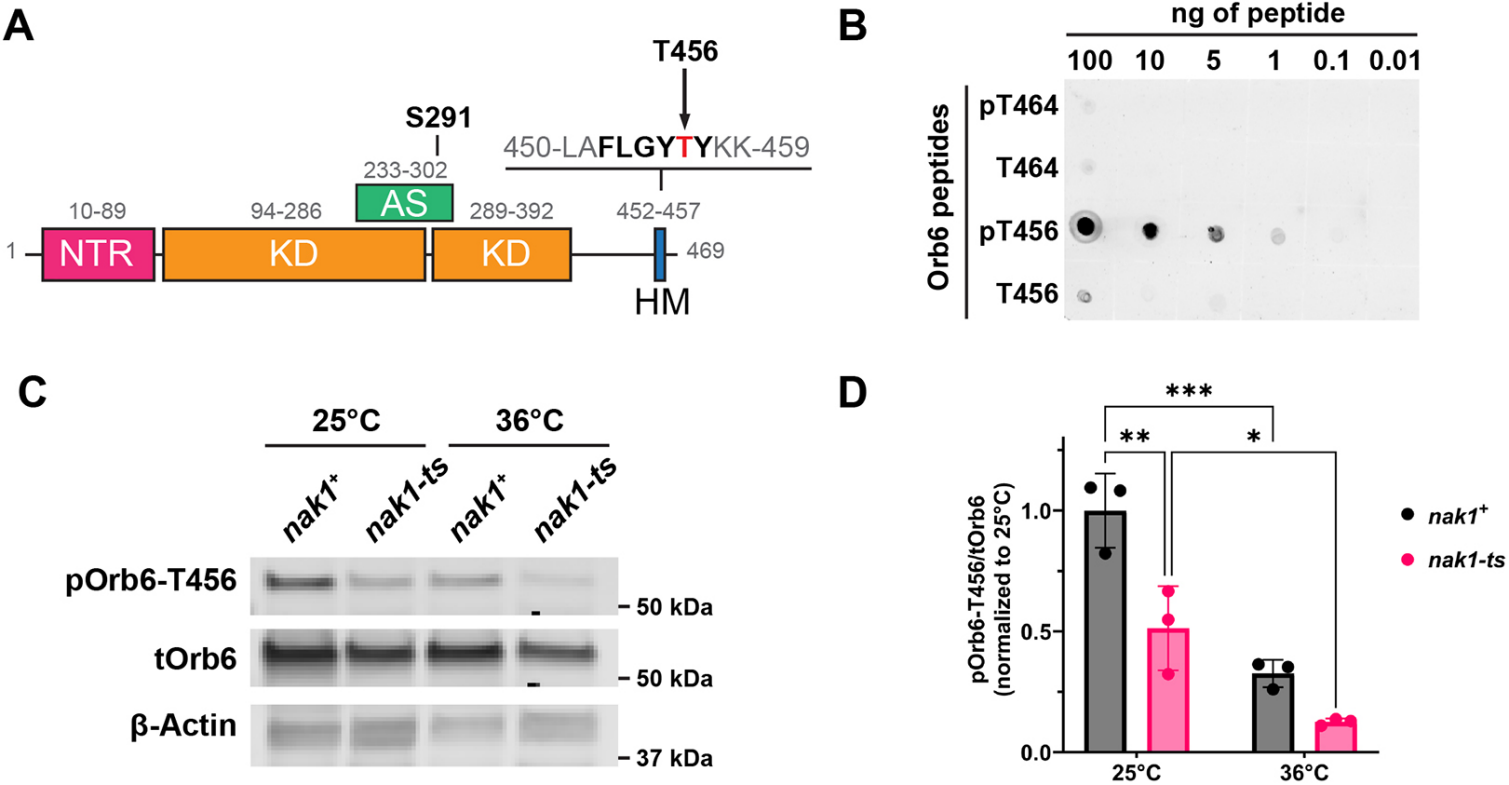
**The phosphorylation state of the Orb6 C-terminal hydrophobic motif is temperature sensitive.** (A) Protein domain map of Orb6 kinase highlighting two phosphosites: S291 in the activation segment and T456 in the hydrophobic motif. NTR, N-terminal region; KD, kinase domain; AS, activation segment; HM, hydrophobic motif. (B) Dot plot showing specificity of anti-phospho (p)-Orb6-T456 antibody. Image shown representative of three repeats. (C) Orb6-456 phosphorylation decreases when temperature sensitive mutant of *nak1* (*nak1-ts*) is exposed to restrictive temperature for 3 h. (D) Quantification of pOrb6-T456/tOrb6 from experiments as in C based on three independent experiments. Data are presented as mean±s.d. *P≤0.05, **P≤0.01, ***P≤0.001 (two-way ANOVA with Tukey's HSD test).

To better study the molecular mechanisms of Orb6 kinase regulation, we developed a phospho-specific antibody that recognizes Orb6 phosphorylation by an upstream kinase at the T456, which is in the C-terminal hydrophobic motif site (Fig. 5A). Phosphorylation of the conserved phosphorylation site at T456 is essential for Orb6 function (Liu and Young, 2012). We validated the specificity of the antibody against phospho-Orb6-T456 (hereafter referred to as pOrb6-T456) by performing a dot blot with four different peptides of Orb6. The pOrb6-T456 antibody is specific only to phosphorylated T456 and not to the corresponding unphosphorylated peptide or a T464, which is present in a similar sequence at the C terminal (Fig. 5B). Previous studies have identified interactions between Nak1 kinase and Orb6 kinase suggesting that Nak1 functions upstream of Orb6 (Liu and Young, 2012). However, direct phosphorylation of Orb6 by Nak1 has yet to be reported. To test whether Orb6-T456 phosphorylation is Nak1-dependent, we exposed temperature-sensitive mutant of *nak1* (*nak1-ts/orb3-167*) (Snell and Nurse, 1994; Leonhard and Nurse, 2005) to restrictive temperature (36°C) for 3 h to downregulate Nak1 activity and measured pOrb6-T456 levels in HA-tagged Orb6 (Fig. 5C). We find that pOrb6-T456 strongly decreases at 36°C in the *nak1-ts* mutant (Fig. 5C,D). Furthermore, we find that pOrb6-T456 also decreases to a lesser extent at 36°C in the control *nak1+* cells. (Fig. 5C,D). At the control temperature of 25°C, a decrease in Orb6-T456 phosphorylation is apparent in the *nak1-ts* mutant, which is consistent with previously reported findings of reduced Nak1 kinase activity of the same *nak1-ts* mutant allele (*nak1-ts/orb3-167*) at 25°C (Leonhard and Nurse, 2005). These results indicate the Orb6 phosphorylation at the C-terminal hydrophobic motif T456 site is dependent on Nak1. Our observations also suggest that Orb6-T456 phosphorylation is affected by heat stress.

**Fig. 6.**
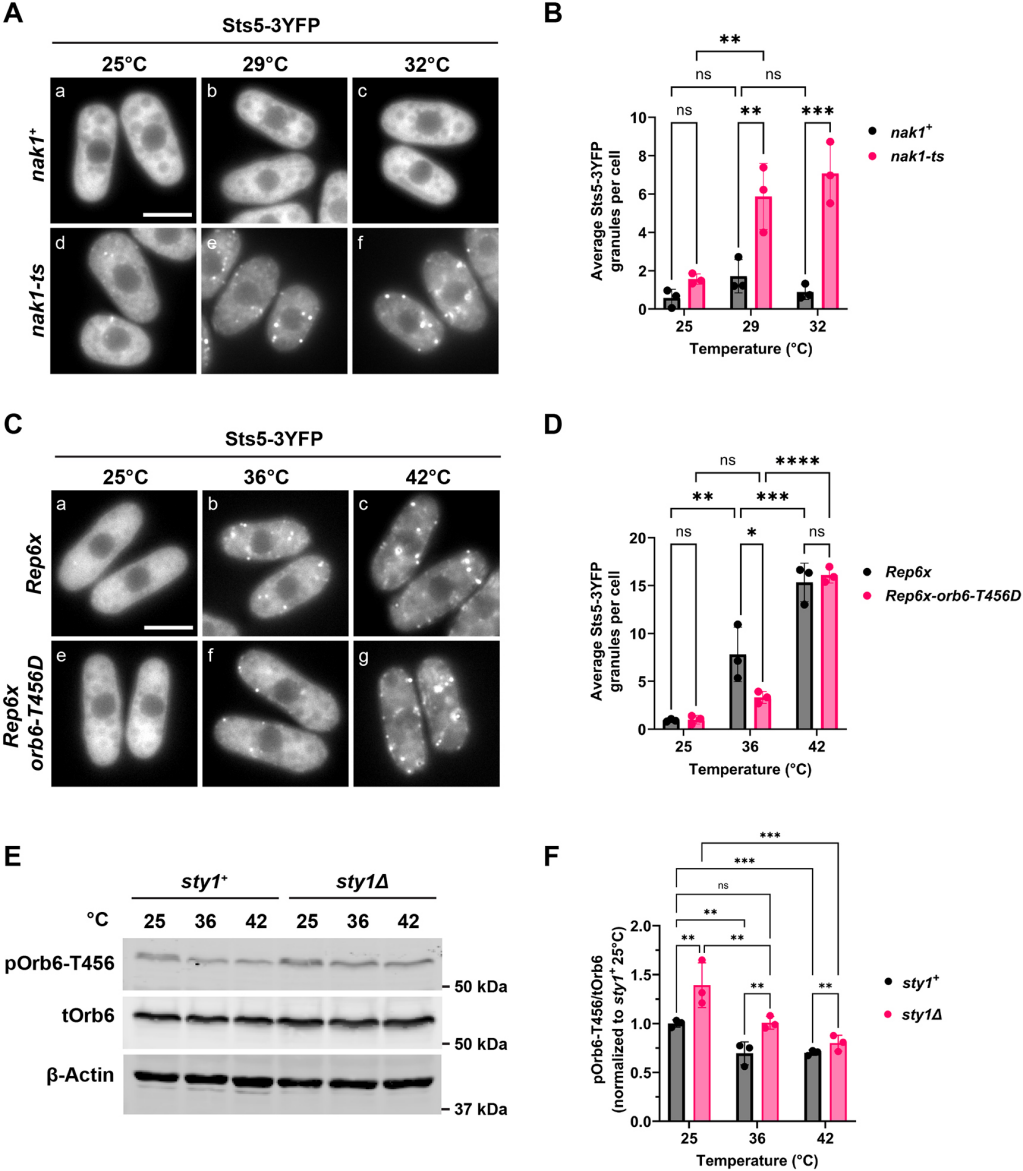
**The phosphorylation state of the Orb6 C-terminal hydrophobic motif is temperature sensitive and regulates Sts5 puncta formation during heat stress.** (A) Sts5–3YFP forms puncta at the semi-permissive temperatures of 29°C or 32°C in the *nak1-ts* mutant and remains diffused in the cytoplasm in the control strain. (B) Quantification of average Sts5–3YFP puncta per cell from experiments as in A based on three independent experiments. Data are presented as mean±s.d. **P≤0.01; ***P≤0.001; ns, not significant (two-way ANOVA with Tukey's HSD test). (C) *orb6-T456D* suppresses Sts5-3YFP granule assembly during heat stress at 36°C but not at 42°C. (D) Quantification of average Sts5-3YFP puncta per cell from experiments as in C based on three independent experiments. Data presented as in B. *P≤0.05, **P≤0.01, ***P≤0.001, ****P≤0.0001; ns, not significant (two-way ANOVA with Tukey's HSD test). (E) Orb6-T456 phosphorylation decreases after 30 min at 36°C or 42°C in control or *sty1Δ* deletion mutant cells. Orb6-T456 phosphorylation is higher in *sty1Δ* mutants. β-actin was used as a loading control. (F) Quantification of pOrb6-T456/tOrb6 from experiments as in E based on three independent experiments. Data presented as in B. **P≤0.01; ***P≤0.001; ns, not significant (two-way ANOVA with Tukey's HSD test). Scale bars: 5 μm.

To investigate the effects of Nak1 kinase and Orb6-T456 phosphorylation on RNP granule assembly, we visualized Sts5–3YFP puncta formation in the *nak1* temperature-sensitive mutant (*nak1-ts*) at the semi-permissive temperatures of 29°C and 32°C for 30 min. We found that the *nak1-ts* mutants readily formed Sts5–3YFP puncta at 29°C and 32°C whereas Sts5–3YFP remained diffusely localized in the cytoplasm in the control strain (Fig. 6A,B). No changes in SG assembly were observed (data not shown).

To further explore the role of Orb6-T456 phosphorylation on Sts5–3YFP granule assembly, we used a constitutively active mutant of *orb6* (*orb6-T456D*), where T456 is replaced by an aspartic acid residue. We have previously shown that this mutant leads to an increase in Orb6 kinase activity and to a decrease in Sts5–3YFP puncta formation upon nitrogen starvation as compared to the control strain (Chen et al., 2019). Here, we exposed cells to heat stress at 36°C or 42°C for 30 min and found that the *orb6-T456D* mutation leads to decreased Sts5–3YFP puncta formation at 36°C but not at 42°C (Fig. 6C,D). Together, these data indicate that phosphorylation of Orb6-T456 by Nak1 activates Orb6 kinase, repressing Sts5–3YFP puncta formation, whereas dephosphorylation of Orb6-T456 during heat stress promotes Sts5–3YFP granule assembly.

Finally, to test whether Sty1 kinase has a role in modulating Orb6-T456 phosphorylation, we exposed cells to 36°C or 42°C for 30 min in the presence or absence of *sty1.* We found that pOrb6-T456 phosphorylation decreases upon exposure to heat, in both control *sty1^+^* or *sty1Δ* deletion mutants (Fig. 6E,F). However, loss of *sty1* leads to overall increased levels of pOrb6-T456, as compared to control cells (Fig. 6E,F). This increase is particularly evident at 25°C and 36°C, indicating that the presence of Sty1 affects the levels of Orb6 phosphorylation at the T456 hydrophobic motif site at these temperatures. Our observations suggest that the increased levels of Orb6 phosphorylation, in the absence of Sty1, play a role in maintaining sustained Orb6 kinase activity during heat stress at 36°C, thereby suppressing the onset of ectopic Cdc42 activation and the induction of Sts5 granule formation. This idea is supported by the observation that the extent of Gef1-S112 phosphorylation (a readout of Orb6 kinase activity) is similar in *sty1Δ* cells at 36°C and unstressed wild-type controls at 25°C. As Orb6 T456 phosphorylation decreases in response to heat in both control and *sty1Δ* deletion mutant cells, our data also point to a Sty1-independent mechanism of downregulation of Orb6 activity in response to increasing heat stress.

## DISCUSSION

### The activity of the conserved NDR kinase Orb6 is thermosensitive

Proper cellular response to environmental fluctuations, such as heat exposure, is crucial for survival and is increasingly recognized as a key factor in human disease. Heat stress disrupts essential cellular processes, including cell polarization (Vjestica et al., 2013; Pawlik et al., 2013; Singh et al., 2016; Delley and Hall, 1999) and protein translation (Kedersha et al., 2005; Grousl et al., 2009). However, the underlying mechanisms mediating these effects are not completely understood.

**Fig. 7.**
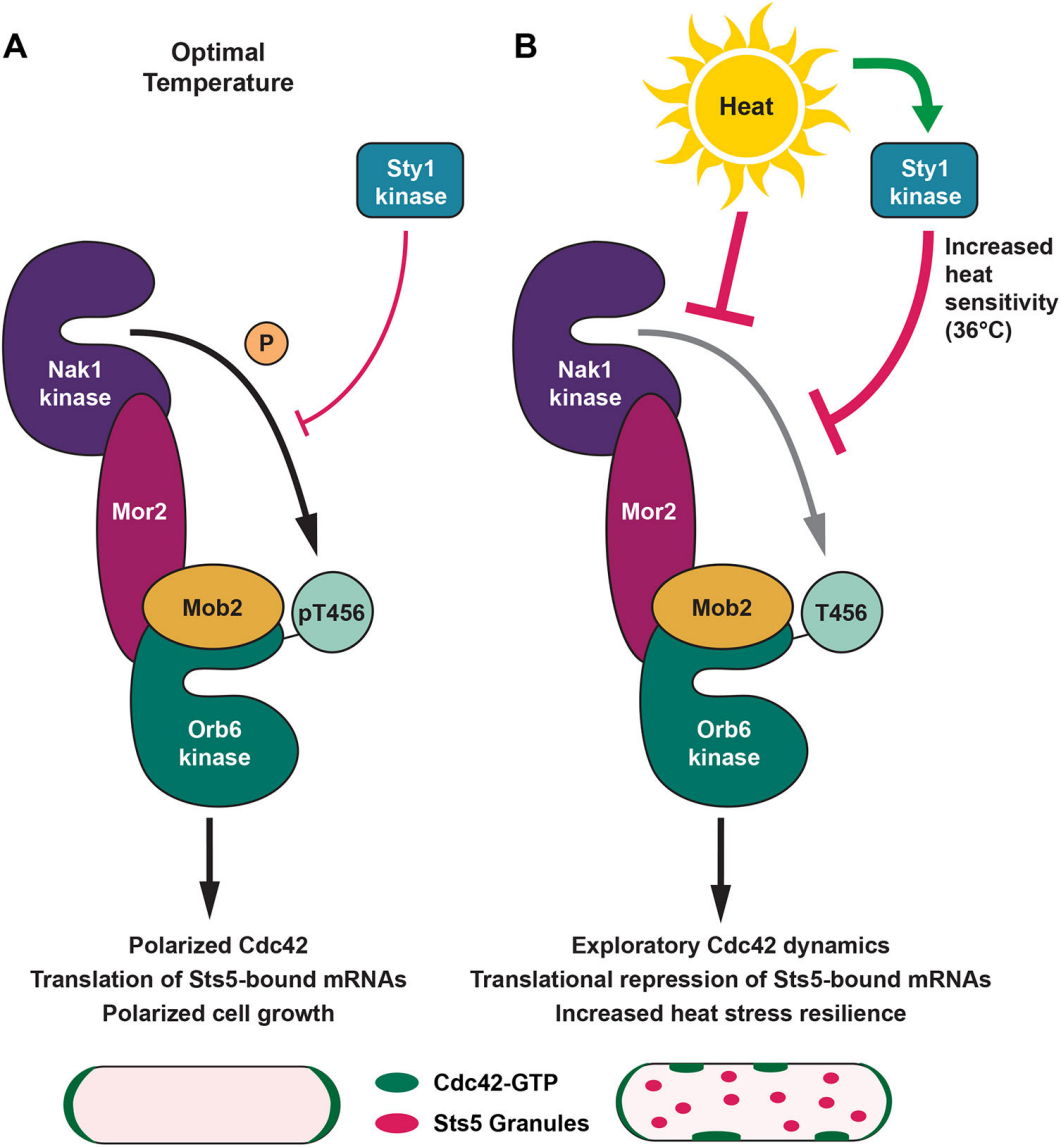
**Regulation of the NDR kinase Orb6 by temperature and MAPK Sty1.** (A) Orb6 kinase activity at optimal, but not stressful temperatures (25°C), promotes the canonical Cdc42 polarity module (via regulation of RBP Sts5) and polarized cell growth. (B) Upon heat stress at 36°C, MAPK Sty1 activation inhibits Orb6 kinase activity, thereby promoting ectopic Cdc42 activation, Sts5 granule assembly, translational repression of Sts5-associated mRNAs and increased heat stress resilience. The induction of ectopic Cdc42 activation also involves direct phosphorylation of Orb6 substrates by Sty1 kinase (see Discussion). Inhibition of Orb6 kinase activity during heat stress also engages Sty1-independent regulatory mechanisms, likely affecting other components of the MOR (morphogenesis Orb6 network) pathway. The activity of MAPK Sty1 lowers the threshold temperature that triggers Orb6 inhibition, thereby rendering Orb6 more temperature sensitive and promoting thermotolerance.

Here, we demonstrate that the conserved fission yeast NDR kinase Orb6, homologous to mammalian STK38, is inactivated by heat stress, altering the distribution of active Cdc42 at the membrane and facilitating RNP granule assembly. We further uncover that the stress-activated MAPK Sty1 negatively regulates Orb6 activity and Orb6 C-terminal phosphorylation during heat exposure (Fig. 7). This inhibition sensitizes cells to temperature-specific heat stress and promotes thermotolerance. These findings underscore the pivotal role of NDR kinase signaling in cellular adaptation to elevated temperatures and reveal a novel role for conserved stress activated MAPK Sty1 in RNP granule formation, mediated by Orb6 kinase. As NDR kinases are strongly conserved, these principles are likely generalizable beyond yeast.

### Orb6 downregulation fosters the emergence of ectopic Cdc42 activation during heat stress

Cdc42 is a conserved Rho-family GTPase that controls polarity, actin organization, vesicle trafficking and exocytosis. In mammalian myoblasts, heat stress reduces migration, at least in part through *CDC42* downregulation (Lu et al., 2023). In fission yeast, active Cdc42-GTP oscillates between tips to drive polarized growth (Das et al., 2012), but under environmental stress (including heat) Cdc42 exhibits ectopic activation along the membrane, deviating from tip-localized oscillations (Vjestica et al., 2013; Chen et al., 2019; Salat-Canela et al., 2021; Haupt et al., 2018). Here, we show that mild and severe heat stress inhibit Orb6 kinase. We previously found that Orb6 inhibition causes dephosphorylation of Cdc42 regulators Gef1 and Rga3 and their release from 14-3-3 protein Rad24 (Das et al., 2015; Doyle et al., 2025), thereby switching on a stress-activated Cdc42 module that includes Gef1 and Rga3 (Salat-Canela et al., 2021; Doyle et al., 2025). Our findings provide a mechanistic explanation for ectopic Cdc42 activation during heat stress. Functionally, alternative Cdc42 dynamics might help cells transition through stress – during nitrogen deprivation they may facilitate mating partner search (Bendezú and Martin, 2013). In other stresses, the engagement of the MAPK–Orb6 regulatory axis (Doyle et al., 2025) could enable transient polarity loss, then fostering the re-establishment of new growth sites upon recovery (Mutavchiev et al., 2016), whereas polarity disruption together with translational repression (see below) might preserve energy (Salat-Canela et al., 2023) and extend lifespan (Chen et al., 2019).

### Sts5 assembles into RNP granules and promotes thermotolerance

Upon exposure to a variety of external stressors, cells form SGs that help promote stress resilience and survival. The RBP Sts5 is highly conserved in eukaryotes and related to human Dis3L2. In fungi, Sts5 is closely related to *Saccharomyces cerevisiae* and *Candida albicans* Ssd1 and *Neurospora crassa* GUL-1 (Astuti et al., 2012; Lv et al., 2015; Robinson et al., 2015; Malecki et al., 2013; Toda et al., 1996; Vaggi et al., 2012; Jansen et al., 2009; Kurischko et al., 2011). These RNA-binding regulators finetune translation of cell wall and morphogenesis-related genes, crucial for stress adaptation, survival and pathogenicity. In *S. pombe* and *S. cerevisiae*, Sts5 and Ssd1, respectively, bind to 5′ UTRs of mRNAs encoding cell wall proteins, repressing their translation to control cell wall biogenesis (Nuñez et al., 2016; Hogan et al., 2008). In fission yeast, Sts5 shapes the translational landscape of mRNAs involved in polarized cell growth and Cdc42 activity, regulating the Ras1-Scd1-Cdc42 regulatory axis (Chen et al., 2019; Nuñez et al., 2016). By promoting translational repression during nutritional stress, both Sts5 and Ssd1 influence entry into and recovery from quiescence (Chen et al., 2019; Miles et al., 2019). Both Sts5 (this paper) and Ssd1 (Mir et al., 2009) are essential for tolerance to heat. Thus, the deep conservation of structure and cellular function through such enormous evolutionary distance highlights the crucial role of these RBPs in cell adaptation and survival.

A shared feature of these proteins is stress-induced LLPS driven by N-terminal intrinsically disordered regions (IDRs). The N-terminal domain is crucial to mediate LLPS of Sts5, Ssd1 and GUL-1 into RNP granules, and is phosphorylated by conserved NDR kinase Orb6 in *S. pombe* (Chen et al., 2019), Cbk1 in *S.s cerevisia*e (Kurischko et al., 2011; Jansen et al., 2009) and COT-1 in *N. crassa* (Stein et al., 2020) and other kinases [such as *S. pombe* Pak1 (Magliozzi and Moseley, 2021)]. We have previously shown that serine 86 in the N-terminus of Sts5 mediates binding to the 14-3-3 protein Rad24 and prevents granule formation; this association maintains active translation of Sts5-associated RNAs (Chen et al., 2019; Nuñez et al., 2016). Conversely, the *sts5-S86A* mutation prevents phosphorylation at that site, inhibits Rad24 binding, promotes RNP granule assembly and decreases translation of Sts5-regulated RNAs (Chen et al., 2019). Subtle enhancement of RNP assembly by Sts5-S86A increases cellular resilience to stress (Chen et al., 2019). Here, we show Sts5-S86A also boosts heat resilience, whereas loss of *sts5* increases heat vulnerability. We have previously shown that Sts5 reduces the availability of specific transcripts for translation (e.g. *ssp1* and *efc25* mRNAs) (Nuñez et al., 2016). Consistent with translational repression during heat exposure, Ssp1 protein levels increase markedly upon heat stress when *sts5* is deleted (Nuñez et al., 2016).

P-bodies and SGs are dynamic cytoplasmic RNP assemblies that regulate mRNA, assembling rapidly during stress and dispersing upon recovery. P-bodies contain mRNA decay and the decapping enzymes Dcp1 and Dcp2, which function in mRNA storage and translational repression; P-bodies exist under basal conditions but increase in size and number during stress (Sakuno et al., 2004; Ingelfinger et al., 2002; van Dijk et al., 2002; Wang et al., 2002). SGs form when translation initiation is inhibited (e.g. by heat or oxidative stress), often via phosphorylation of eIF2α and inhibition of the pre-initiation complex, thereby storing untranslated mRNAs for later re-entry into translation (Marcelo et al., 2021; Panas et al., 2016). Although P-bodies and SGs are compositionally distinct, both assemble initially through LLPS, with RBP coalescence thought to nucleate granules (Aguzzi and Altmeyer, 2016; Kroschwald et al., 2015; Lin et al., 2015; Elbaum-Garfinkle et al., 2015; Patel et al., 2015; Kato et al., 2012; Hyman et al., 2014; Brangwynne et al., 2009; Lee et al., 2013; Brangwynne, 2013; Becker and Gitler, 2015). Importantly, P-bodies and SGs interact physically and functionally, exchanging components and mRNAs during stress and recovery, which might finetune global translation rates. The kinetics of assembly and dissolution are reversible on the timescale of minutes to hours, offering cells a rapid, energy-efficient means to reprogram translation. In our system, the degree of Sts5 co-partitioning with P-bodies versus SGs changes at 25°C, 36°C and 42°C, suggesting temperature-dependent routing of mRNAs between storage, decay and re-initiation pools.

During nitrogen or glucose deprivation, Sts5 forms puncta partially colocalizing with the P-body marker Dcp1–mCherry (Chen et al., 2019; Nuñez et al., 2016). Here, we find that mild heat stress (36°C) similarly induces Sts5 granules that colocalize with Dcp1, whereas Pabp1-containing SGs are not detectable. Under severe heat stress (42°C), when visible SGs form, Sts5 colocalizes with Pabp1. Consistent with P-bodies and RBPs promoting stress-granule assembly (Buchan et al., 2008; Wheeler et al., 2016), loss of *sts5* greatly reduces the number of SGs and decreases cell survival during heat shock. The gain in thermos-resilience is greater for Orb6 downregulation than for *sts5-S86A* mutant, implying Orb6 also has Sts5-independent roles in thermotolerance.

Orb6 inhibition alone is not sufficient to induce stress-granule formation, indicating additional regulatory inputs. For example, other factors such as the vigilin homolog Vgl1 (Wen et al., 2010), signaling factors such as calcineurin (Higa et al., 2015) and protein kinase C Pck2 (Kanda et al., 2021) also associate with SGs in response to thermal stress, and SG formation is also stimulated in response to heat stress by the RBP Nrd1 (Satoh et al., 2012). These likely act in parallel with Sts5 to drive robust granule nucleation and maturation and might integrate upstream stress signals with translational control.

### The MAPK Sty1-NDR kinase Orb6 regulatory axis regulates Cdc42 dynamics and RNP granule assembly during heat stress

Sty1 is the stress-activated MAPK (SAPK) in fission yeast, analogous to mammalian p38 or JNK proteins. Heat stress transiently activates Sty1 through its dual phosphorylation by Wis1 at two conserved residues, T171 and T173, and leads to Sty1 nuclear accumulation (Degols et al., 1996; Smith et al., 2002). Under basal unstressed conditions, Sty1 activity is negatively regulated by tyrosine phosphatase Pyp1; upon heat stress, thermosensitive Pyp1 phosphatase misfolds, leading to impaired Sty1 interaction and increased Sty1 kinase activity (Nguyen and Shiozaki, 1999; Boronat et al., 2020). Sty1 activation during heat stress stabilizes the tyrosine phosphatase Pyp2, highlighting a self-regulatory mechanism by Sty1 to return to basal conditions (Kowalczyk et al., 2013). *Sty1Δ* mutants are severely thermosensitive (Yamada et al., 1997), and Sty1 activation is required for survival both at 37°C (Yamada et al., 1997) and at ≥42°C (Berlanga et al., 2010; Yamada et al., 1997; Degols et al., 1996). Beyond acute signaling, Sty1 drives transcriptional programs that remodel stress responses, potentially intersecting with the translational and RNP granule-based controls described here.

Our results show that Orb6 activity decreases during mild and severe heat stress. We recently reported that Sty1 negatively regulates Orb6 during nitrogen starvation (Doyle et al., 2025), providing a mechanistic understanding for Sty1-dependent ectopic Cdc42 activation that might facilitate mating (Bendezú and Martin, 2013; Merlini et al., 2016). Sty1 inhibits Orb6, releasing the Orb6 substrates Gef1 and Rga3 from the 14-3-3 protein Rad24 (Doyle et al., 2025), and directly phosphorylates both regulators, likely enhancing their activity (Salat-Canela et al., 2021). This creates a coherent feedforward loop that promotes ectopic Cdc42 activation during stress (Salat-Canela et al., 2021; Doyle et al., 2025). Although this mechanism of Gef1 regulation is crucial, other kinases might also regulate Gef1 activity and function. Namely, the mitotic regulator Dsk1 kinase has been shown to modify Gef1 in a phosphoproteomics approach, although the functional consequences of this phosphorylation are unclear (Wu et al., 2020).

Here, we show that the Sty1-Orb6 axis also functions during heat stress. Through Orb6, Sty1 modulates the phase-separation properties of Sts5 and thereby the coalescence of P-bodies and SGs in response to heat. Notably, loss of Sty1 completely suppresses the heat-dependent effects of Orb6 inhibition, that is, ectopic Cdc42 activation, Gef1 dephosphorylation and Sts5 aggregation, at 36°C, but not at 42°C. Experimentally, these findings indicate a temperature-specific control of Orb6 by Sty1 that is effective at sublethal 36°C but not at 42°C.

### The role of the C-terminal hydrophobic motif containing Orb6 T456 in thermal sensation

AGC-family kinases contain a conserved C-terminal hydrophobic motif with a phosphorylatable serine or threonine residue (in Orb6, T456). Phosphorylation of Orb6-T456 is essential for stabilizing the active conformation of the kinase, facilitating proper alignment of catalytic residues for efficient substrate phosphorylation, and strongly correlates with kinase activity (Millward et al., 1999; Hergovich et al., 2006; Hergovich, 2013; Jansen et al., 2006; Brace et al., 2011; Parker et al., 2020).

We show that Orb6 phosphorylation at T456 decreases as temperature increases. Upon loss of Sty1, the overall levels of Orb6-T456 phosphorylation substantially increase, an effect that is particularly striking at 25°C and 36°C, indicating that Sty1 has a role in the negative control of Orb6-T456 phosphorylation. However, Orb6-T456 dephosphorylation during heat occurs even in *sty1Δ* cells, suggesting that a Sty1-independent mechanism also contributes to Orb6 T456 regulation. In *sty1Δ* cells at 36°C, T456 phosphorylation levels are comparable to those in wild-type cells at 25°C; consistent with sustained Orb6 activity, Gef1 phosphorylation, Cdc42 distribution and Sts5 localization remain largely unperturbed, and cells are less responsive to heat stress. Sts5–3YFP granule assembly during heat is induced, at least in part, by decreased Nak1 kinase activity and T456 dephosphorylation, linking Orb6 HM phosphorylation to RNP granule assembly. Thus, this finely tuned regulatory network ensures the inhibition of Orb6 during mild heat stress (36°C). This allows the cell to reprogram its translational landscape even in the absence of SG formation and promote morphogenetic changes that may be necessary for survival.

It is unclear what could be the Sty1-independent mechanism of temperature-sensing that leads to Orb6 inactivation. One possible mechanism is that Nak1, the kinase upstream of Orb6, is directly inhibited by heat stress. Another possible mechanism is that heat affects protein structure of the Orb6 regulatory complex, perhaps the association of the co-activator Mob2 to Orb6, or the interaction of the Mor2 scaffold with Nak1 and Orb6. Direct inhibition of the Mob2–Orb6 complex by an inhibitory protein is also possible, as detailed for the *S. cerevisiae* protein Lre1, which does not however have homologs in *S. pombe* or higher eukaryotes (Mancini Lombardi et al., 2013; Versele and Thevelein, 2001). Other potential candidates could be growth control pathways like phosphoinositide 3-kinase (PI3K), which promotes SG assembly in a hierarchical manner with p38 MAPK in human cells (Heberle et al., 2019). In *S. pombe*, Pik3 is the conserved homolog of human PI3K and loss of *pik3* abolishes cell growth at higher temperatures (Koga et al., 2004), indicating an important role of Pik3 in cell survival during heat stress. Other prospective pathways that could be involved in the Sty1-independent regulation of Orb6 are cell polarity signaling regulator Pak1 (Verde et al., 1998) or glucose responsive pathway Pka1, both of which have been found to regulate SG assembly in fission yeast (Magliozzi and Moseley, 2021; Nilsson and Sunnerhagen, 2011). Future experiments will address these possibilities.

### Conclusion

Our study highlights the role of the MAPK Sty1-NDR kinase Orb6 regulatory axis in promoting thermotolerance during environmental heat exposure. By coordinating Cdc42 dynamics, translational repression and RNP granule assembly, this pathway supports adaptation to elevated temperatures. Given the deep evolutionary conservation of NDR kinases, these mechanisms likely extend to higher eukaryotes and have potential implications for crop survival, pathogen spread and clinical strategies that exploit hyperthermia to improve therapeutic outcomes. Thus, understanding how MAPK and NDR signaling integrate extracellular stress cues with intracellular phase separation, mRNA control, and cell polarization may inform interventions that enhance resilience across biological systems.

## MATERIALS AND METHODS

### Strains and growth medium

*S. pombe* strains used in this study were derived from wild-type strains 972 or 975 and are displayed in Table S1. *S. pombe* was maintained in yeast extract plus supplements (YES; Sunrise Science, cat. no. 2011) or Edinburgh minimal medium (EMM) supplemented with 0.5% ammonium chloride (EMMN; Sunrise Science, cat. no. 2005) and additional supplements (Sigma) as needed [histidine (cat. no. H8125), leucine (cat. no. L8912), adenine (cat. no. A8751) and/or uridine (cat. no. U3750)] at a concentration of 225 mg/l. All strains were cultured at 25°C unless otherwise noted. Liquid cultures were incubated at 180 rpm in a shaking incubator, and all cultures were diluted daily such that they were maintained in logarithmic growth for a minimum of eight generations prior to performing experiments.

### Fluorescence microscopy

For experiments utilizing microscopy, samples were imaged with an Olympus BX61 fluorescent microscope using appropriate filters. Exposure times range from 1000–2500 ms for each experiment and remain consistent among all samples for that experiment. Images were acquired and processed using Intelligent Imaging Innovations SlideBook image analysis software (Version 6.0.4; Denver, CO) and prepared with Fiji (Schindelin et al., 2012). Binucleated mitotic cells or cells with a septum were omitted from quantification as Orb6 activity has been shown to be downregulated (Kanai et al., 2005) and Sts5 forms clusters during mitosis (Vaggi et al., 2012).

### RNP granule induction

To determine localization of Sts5 puncta relative to SGs, we introduced the marker Pabp1–DsRed, constructing a *sts5-3YFP pabp1-DsRed* strain (FV3194). We tested the localization of Sts5 relative to SGs upon heat stress at 36°C or 42°C for 30 min. To determine whetehr Sts5 was required for the formation of SGs, *sts5^+^ pabp1-DsRed* (FV1684) and *sts5Δ pabp1-DsRed* (FV3192) mutants were exposed to heat stress at 42°C for 30 min or glucose limitation (see below). Images are representative of samples from three independent experiments.

### RNA fluorescent *in situ* hybridization

Localization of *ssp1* mRNA was determined using RNA fluorescent *in situ* hybridization (RNA-FISH) as previously described, with modifications according to Nunez et al. (Nuñez et al., 2016; Nilsson and Sunnerhagen, 2011; Heinrich et al., 2013; Brengues and Parker, 2007). *sts5-3YFP pabp1-DsRed* cells (FV3194) were cultured in EMMN with required supplements and exposed to 42°C for 30 min or left at 25°C as a control prior to fixation and hybridization of RNA was performed with 20-mer DNA oligonucleotides (Stellaris) labeled with Quasar 705 fluorochromes.

### Glucose starvation

For glucose limitation, samples were cultured to log phase in EMMN containing 2% glucose and were washed once with 5 ml EMMN lacking glucose. Cells were then resuspended in 5 ml EMMN lacking or containing glucose and were incubated at 25°C and 180 rpm in a shaking incubator for 20 min. Following incubation, 1 ml aliquots were centrifuged at 1500 ***g*** for 1 min and resuspended in a small amount of residual medium. Microscopy was performed as described above and the number of SGs was manually counted. This experiment was performed in triplicate, and *n*=90 cells per strain per condition in total.

### Heat-shock survival assay

Wild-type (FV2644), *sts5Δ* (FV2674), *sts5-HA* (FV2645) and *sts5-S86A-HA* (FV2649) strains were cultured in EMM and maintained in log-phase for a minimum of eight generations. Each strain was diluted to an optical density at 595 nm (OD_595nm_)=0.2 (∼4×10^6^ cells/ml) in 5 ml EMM and allowed to grow at this density for 1 h at 25°C shaking at 180 rpm. Samples were either maintained at 25°C or heat shocked at 48°C for 15 min. Following heat shock, experimental and control samples were each serially diluted, plated in triplicate on EMM plates and incubated at 25°C for approximately 72 h. Colony forming units per ml (CFUs/ml) were calculated for each condition, and percentage survival was calculated by dividing CFUs/ml for heat-shocked replicates by their respective untreated control CFUs/ml. Percentage survival of each technical replicate was normalized to the average untreated control percent survival. The average percentage survival for each strain is as follows: wild-type, 1.8%; *sts5Δ*, 0.2%; *sts5-HA*, 1.3%; and *sts5-S86A-HA*, 4.0%. This experiment was performed in biological triplicate, and data was normalized to the average wild-type percentage survival for each independent experiment and subsequently graphed as the relative survival rate. To determine the impact of intermediate to long-term restrictive temperature on these strains, this experiment was repeated with modifications. In this case, samples were incubated at 25°C or 36.5°C for 6 h. Following incubation, cells were serially diluted and plated in triplicate, and incubated at 25°C for ∼72 h. Calculation of CFUs/ml and normalization of data was performed as previously described, and this experiment was performed in biological triplicate. The average percentage survival for each strain (normalized to the control at 25°C) is as follows: wild-type, 125.6%; *sts5Δ*, 65.2%; *sts5-HA*, 120.8%; and *sts5-S86A-HA*, 179.9%.

For heat-shock survival analysis of the *orb6-as2* strain (FV2527) and its respective wild-type control (FV2530), cells were initially pre-cultured in minimal medium supplemented with adenine at 180 rpm and 32°C. In order to repress the expression of *orb6-as2* the culture was split into two sets, either with or without thiamine at a final concentration of 15 µM and was allowed to grow for ∼16 h. Cells were diluted to an equivalent OD_595nm_ of 0.2 (4×10^6^ cells/ml) in appropriate media, and cells were allowed to grow for an additional 2 h. In the absence of thiamine, expression of HA-Orb6-as2 was equivalent to 1.5-fold the Orb6-HA expression under the control of the endogenous *orb6* promoter. Conversely, under these growth conditions in the presence of thiamine, HA-Orb6-as2 protein levels declined by 97.7%, to almost undetectable levels. Samples were then heat shocked in a 48°C shaking water bath for 15 min while controls were maintained at 32°C. All samples were then serially diluted and plated on minimal medium supplemented with adenine, but lacking thiamine, for subsequent quantification of CFUs/ml as described above. The average percentage survival for each strain is as follows: wild-type −thiamine, 0.1%; wild-type +thiamine, 0.1%; *orb6-as2* −thiamine, 0.1%; and *orb6-as2* +thiamine, 18.9%.

### Temperature dependent analysis of Sts5 puncta

*sts5-3YFP* (FV2518) and *sts5-S86A-3YFP* (FV2522) strains were used to determine whether Sts5 puncta are induced at lower temperatures when Sts5 is hyperactive as compared to a wild-type allele. Each strain was cultured to log-phase in EMM, diluted to OD_595nm_=0.1, and cells were allowed to grow for a minimum of 1 h at 25°C. Aliquots of each strain were created, and samples were heat shocked at 29°C, 32°C, 35.5°C or 42°C for 30 min. Control cells were maintained at 25°C throughout the experiment. Following exposure to heat shock, samples were imaged using an Olympus BX61 microscope. To determine differences in number of puncta, 20 cells per strain were manually scored for Sts5–3YFP puncta at each temperature.

### Development of anti-pOrb6-T456 phospho-specific antibody

The anti-pOrb6-T456 phospho-specific antibody was custom-made by 21st Century Biochemicals against a synthetic peptide corresponding to the phosphorylated Thr-456 (amino acids 449-462, NLAFLGYT*YKKFNY) of the fission yeast Orb6 protein. To detect pOrb6-T456, we used a strain expressing N-terminally tagged HA-Orb6 (*HA-orb6-as2*), integrated in single copy behind the *nmt1* promoter, given that increased expression of Orb6 allows for better detection of pOrb6-T456, as compared to C-terminally tagged Orb6-HA or endogenous Orb6 where the detection signal is too low.

### Protein extraction and western blot analysis

Protein extraction was modified from a previously described protocol (Matsuo et al., 2006) and performed as previously described (Doyle et al., 2025). ∼7.5×10^7^ total cells growing exponentially were harvested and cell pellets were resuspended in 300 μl of H_2_O with protease and phosphatase inhibitor cocktail (Halt™ Protease and Phosphatase Inhibitor Cocktail (100×)) and transferred to 1.5 ml microcentrifuge tube. 300 μl of 0.6 M NaOH with inhibitor cocktail (see above) was added to the cells and resuspended. Cells were left to incubate for 5 min at room temperature, inverting the tubes two or three times halfway through incubation. Cells were then centrifuged at 2300 ***g*** for 2 min. The supernatant was discarded, and the pellet was resuspended in 75 μl modified SDS buffer (60 mM Tris-HCl pH 6.8, 4% 2-mercaptoethanol, 4% SDS, 5% glycerol) with inhibitor cocktail (see above) and boiled at 98°C for 3 min. Cells were placed on ice and centrifuged at 4°C at 3500 ***g*** for 1 min. 65 μl of the supernatant (protein extract) was collected with 60 μl stored at −80°C immediately, and 5 μl used for downstream protein quantification assay. To quantify protein, the RC DC protein assay kit was used as it is compatible with the concentration of reducing agents and detergents present in the modified SDS buffer.

Standard western blotting procedures were performed as follows: protein was separated using SDS-PAGE and transferred onto a nitrocellulose membrane. The membranes were probed with antibodies of interest and visualized through fluorescent detection on Li-Cor Odyssey CLx. Gef1–3YFP strains were used in detecting total Gef1 protein with anti-GFP [Roche, cat. no.: 11814460001; RRID:AB_390913; dilution factor (DF): 1:1000] and phosphorylated Gef1-S112 was detected using a custom-made previously described antibody (Chen et al., 2019). HA–Orb6as2 strains were used in detecting total Orb6 protein with anti-HA (Biolegend, cat. no. 901501; RRID:AB_2565006; 1:3000) and phosphorylated Orb6-T456 was detected using a custom-made antibody (see above, 1:1000). Anti-β-actin (Abcam, cat. no.: AB8224; RRID:AB_449644; 1:4000) was used as a loading control for all experiments. Two-color detection was used with using LiCor IRDye secondary antibodies anti-rabbit 800CW (Li-Cor, cat. no.: 926-32211; RRID:AB_621843; 1:15,000) for anti-pGef1-S112 or anti-pOrb6-T456 and anti-mouse 680RD (Li-Cor, cat. no.: 102673-408; RRID:AB_10956588; 1:15,000) for anti-GFP (Gef1–3YFP) or anti-HA (HA-Orb6as2). Quantification of the blots was performed using Image Studio software. To quantify, a rectangle was drawn around the signal band of interest and pixel intensity was recorded. To remove background signal, background settings were set to median, segment: top and bottom, border width: 3. With the pixel intensity measurements, a ratio was determined between pGef1-S112 or pOrb6-T456 (fluorescent channel 800) and total Gef1–3YFP or total HA–Orb6as2 (fluorescent channel 700) to measure how much total Gef1–3YFP protein was phosphorylated at S112 or the total HA–Orb6as2 protein is phosphorylated at T456. Data was normalized by creating a ratio with all values with the average ratio of control strains and/or conditions. Similar quantification was performed to measure ratio of Atf1 to β-actin during heat stress in control cells and *sty1* deletion mutants using anti-Atf1 (Abcam, cat. no.: AB18123; RRID:AB_444264; 1:2000) and anti-β-actin (Abcam, 1:4000). Uncropped blots of western blots shown in this article are provided in Figs S6–S10.

### Quantification and statistical analysis

Data are presented as described in legends. Experiments were completed in independent biological triplicates. A two-tailed unpaired Student's *t*-test was used to assess statistical significance between two groups. One-way or two-way analysis of variance (ANOVA) followed by appropriate post hoc test was applied to evaluate the difference between more than two groups (one-way) or more than two groups and more than one condition (two-way). Statistical analyses and visualization were performed with GraphPad Prism 10 (GraphPad Software, San Diego, CA). *P*<0.05 was set as the threshold for statistical significance. Power analyses were performed using G*Power to determine minimum sample size (Faul et al., 2007; Kang, 2021).

## Supplementary Material

10.1242/joces.264507_sup1 Supplementary information
