# Supplementary material for: Temperature-specific regulation of the NDR kinase Orb6 by the MAPK Sty1 to promote heat stress resilience
Source: J Cell Sci. 2026 Apr 22;139(8):jcs264507. doi: 10.1242/jcs.264507 (PMC13143207; doi:10.1242/jcs.264507)
Supplement: Supplementary information [file joces-139-264507-s1.pdf]

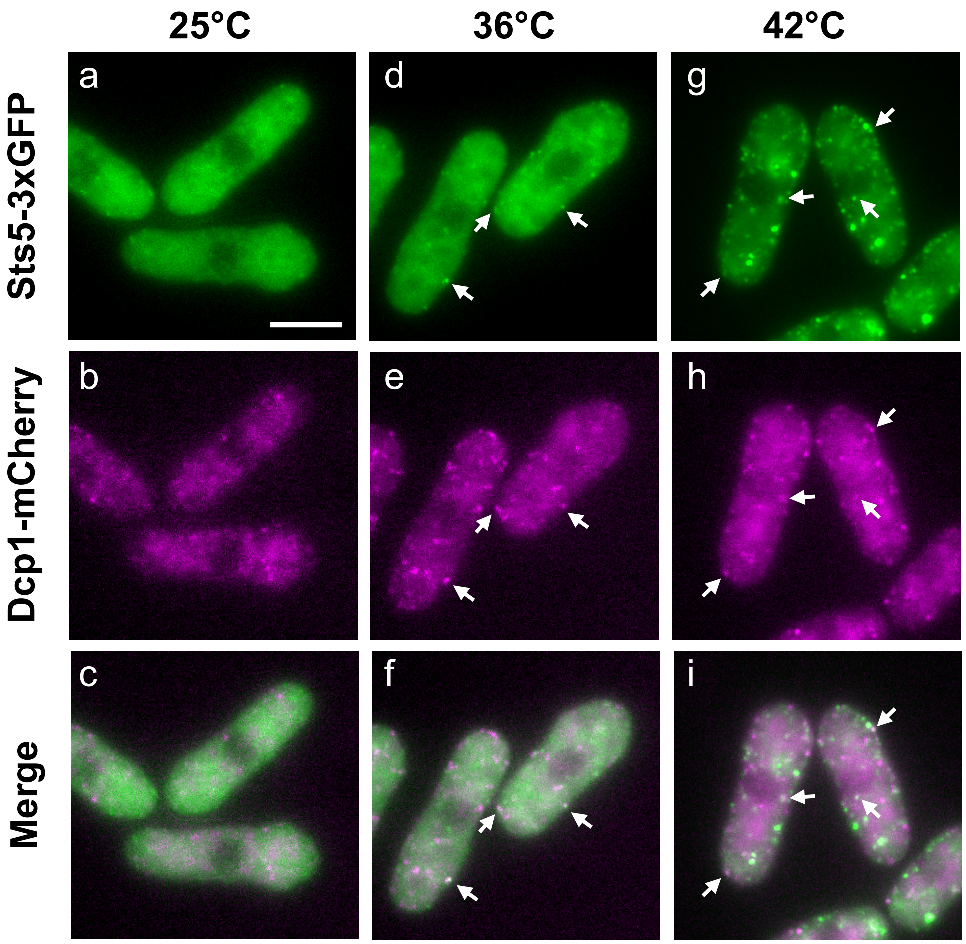

Fig. S1. Sts5-3xGFP co-localizes with P-bodies upon heat stress. Sts5-3xGFP Dcp1-mCherry cells were cultured in YES medium and heat stress at 36ºC or 42ºC for 30 minutes. After heat shock, Sts5-3xGFP formed cytoplasmic puncta which partially co-localized with the P-body marker Dcp1-mCherry at both 36ºC (d-f) and 42ºC (g-i), whereas the cells at control temperature displayed little to no formation of P-Bodies or Sts5 puncta (a-c). Images are deconvolved projections from Z-stacks (6 slices) separated by a step size of 0.3 μM (Bar = 5 μM).

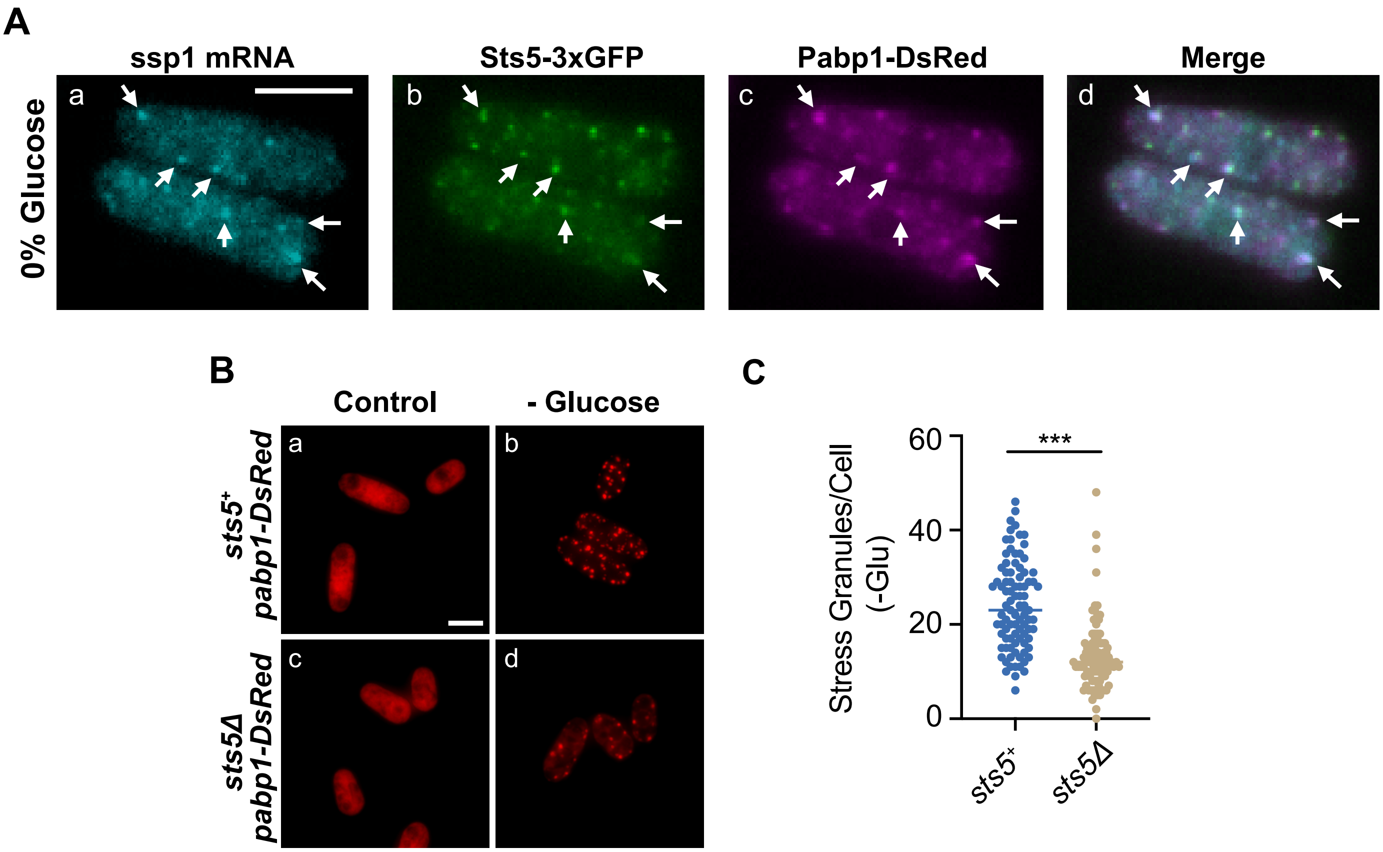

Fig. S2. (A) RNA FISH visualization of *ssp1* mRNA in fixed cells cultured for 20 minutes in minimal medium containing 0% glucose. Hybridization of RNA was performed with 20-mer DNA oligonucleotides (Stellaris) labeled with Quasar 705 fluorochromes. *ssp1* mRNA co-localized with Sts5-3xGFP and the stress granule marker Pabp1-DsRed (arrows). Images are deconvolved projections from Z-stacks (12 slices) separated by a step size of 0.3 μM (Bar = 5 μM). (B) pabp1-DsRed and *sts5Δ pabp1-DsRed cells* were cultured in EMM medium with (a, c) or without (b, d) glucose for 20 minutes. In untreated controls, the stress granule marker Pabp1-DsRed remained diffuse throughout the cytoplasm. Following glucose deprivation, *sts5Δ* cells formed less stress granules than the control (b, d). Representative images are deconvolved projections from Z-stacks separated by a step size of 0.3 μM (Bar = 5 μM). (C) The number of stress granules were quantified for experiment represented in B (n=90 cells/strain/condition total, performed in biological triplicate), and there was a statistically significant decrease in stress granule formation for the *sts5Δ* mutant as compared to the control (***p<0.001; unpaired t-test).

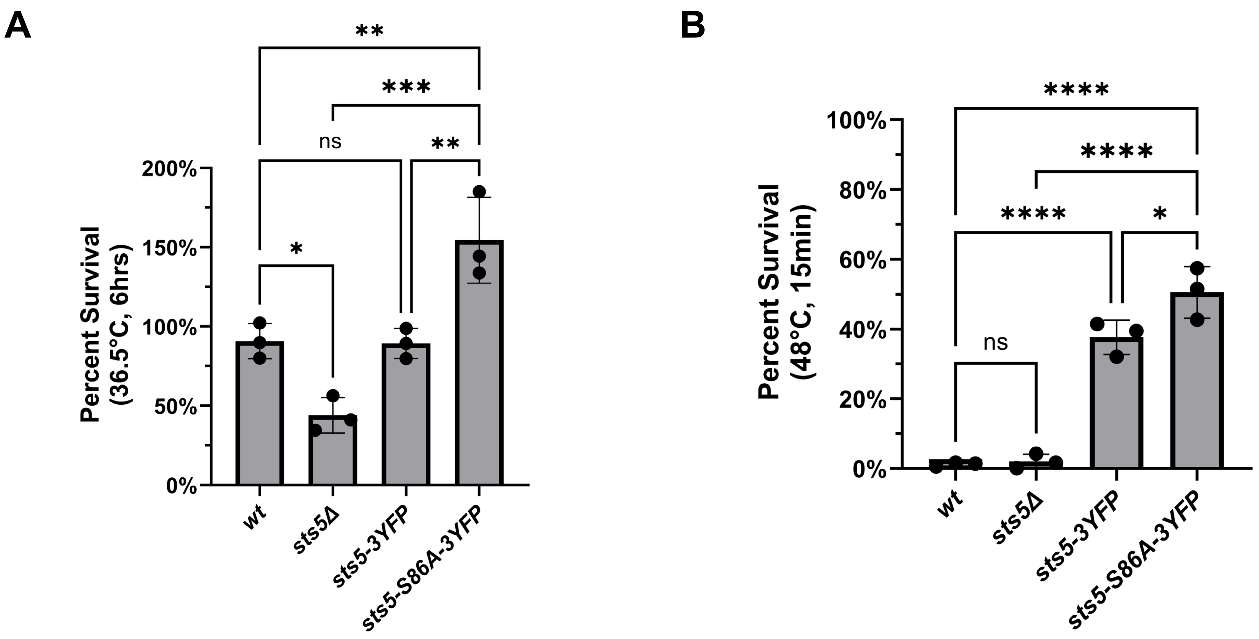

Fig. S3. Sts5 modulates survival after heat stress. (A) Wild-type, *sts5Δ*, *sts5-3YFP*, and *sts5-S86A-3YFP* cells were grown for 6 hours at either 25ºC or 36.5ºC. Following prolonged heat stress at 36.5ºC, *sts5Δ* cells displayed decreased survival compared to wild type, whereas *sts5-S86A-3YFP* showed increased survival compared to the *sts5-3YFP* control based on three independent experiments. Data are presented as mean ± SD, p values determined by one-way ANOVA with Tukey's HSD test $p \leq 0.05$, *; $p \leq 0.01$, **; $p \leq 0.001$, ***. (B) The experiment in A was repeated, but cells were instead incubated for 15 minutes at 48ºC temperature, while untreated controls were incubated at 25ºC. Again, *sts5Δ* cells exhibit decreased survival, whereas the *sts5-S86A-3YFP* mutant exhibited increased survival based on three independent experiments. Data are presented as mean ± SD, p values determined by one-way ANOVA with Tukey's HSD test $p \leq 0.05$, *; $p \leq 0.0001$, ****.

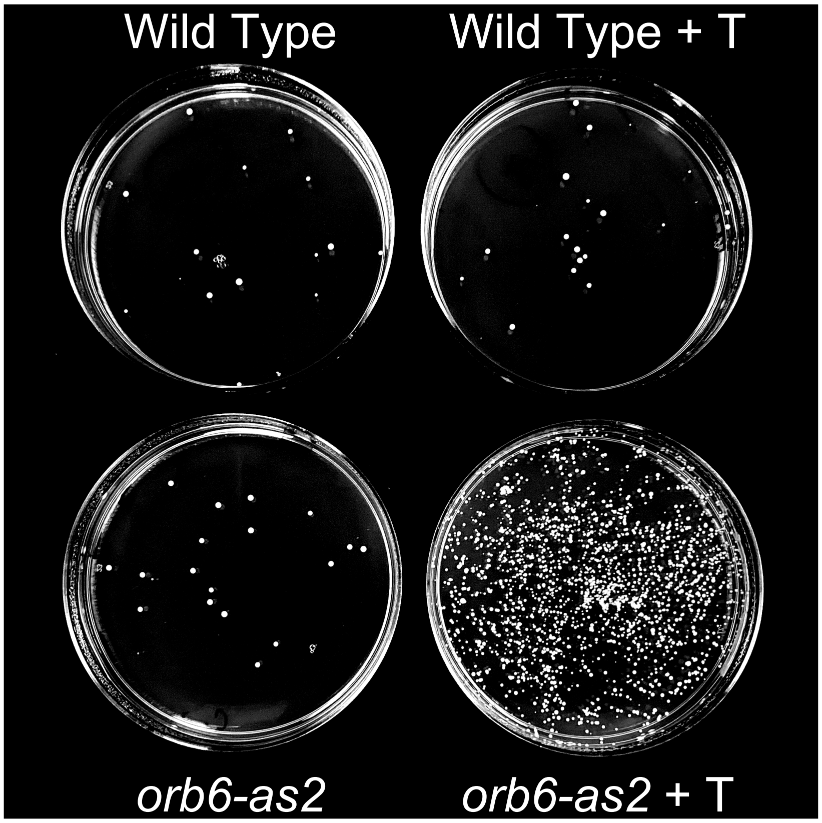

Fig. S4. Downregulation of Orb6 promotes survival after heat shock. Representative image of the orb6-as2 heat shock assay (10-2 dilution) from the heat shocked sample set. Inhibition of Orb6 drastically increases survival (bottom right) after exposure to a 48ºC heat shock for 15 minutes, as compared to untreated or wild-type controls.

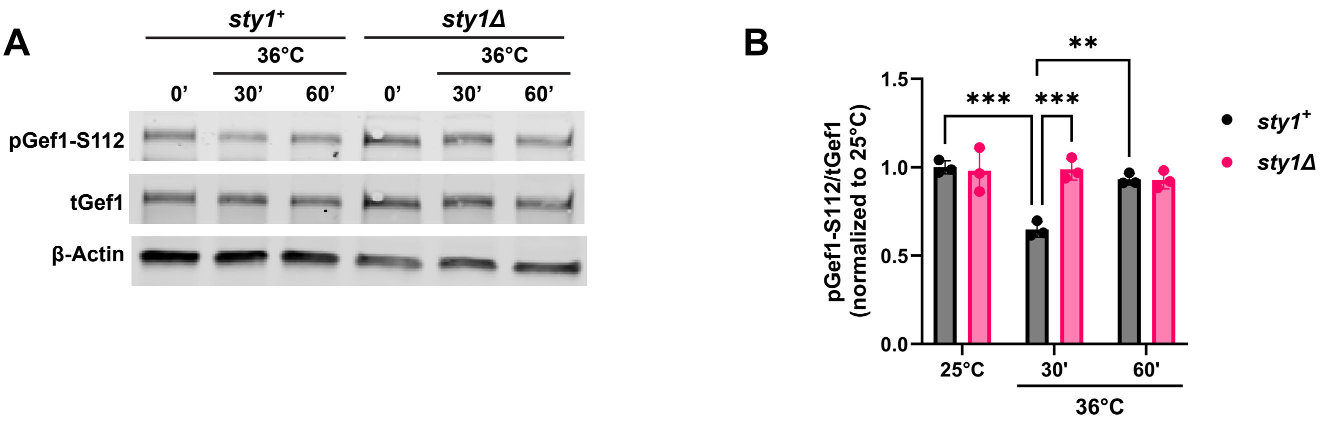

Fig. S5. (A) Gef1-S112 phosphorylation by Orb6 decreases after 30 minutes at 36°C and recovers after 60 minutes. In *sty1Δ* deletion cells, pGef1-S112 remains constant at 36°C at 30 and 60 minutes. β-Actin was used as a loading control. (B) Quantification of pGef1-S112/ tGef1 from A upon temperature stress exposure in control or *sty1Δ* deletion mutant cells based on three independent experiments. Data are presented as mean ± SD, p values are determined by two-way ANOVA with Tukey's HSD test $p \leq 0.01$, **; $p \leq 0.001$, ***.

Journal of Cell Science • Supplementary information

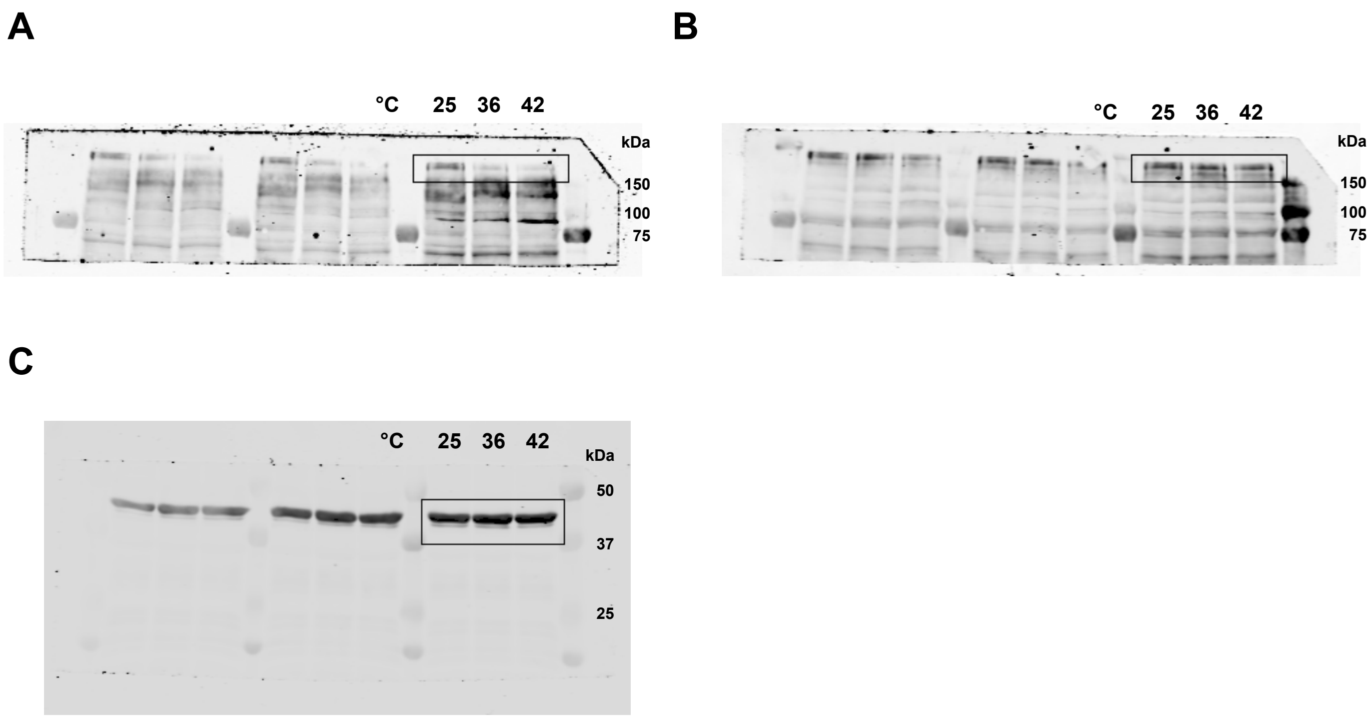

Fig. S6. Uncropped blots from Fig. 1C. (A) anti-pGef1-S112. (B) anti-GFP (total Gef1-3YFP). (C) anti-Actin for A-B.

Journal of Cell Science • Supplementary information

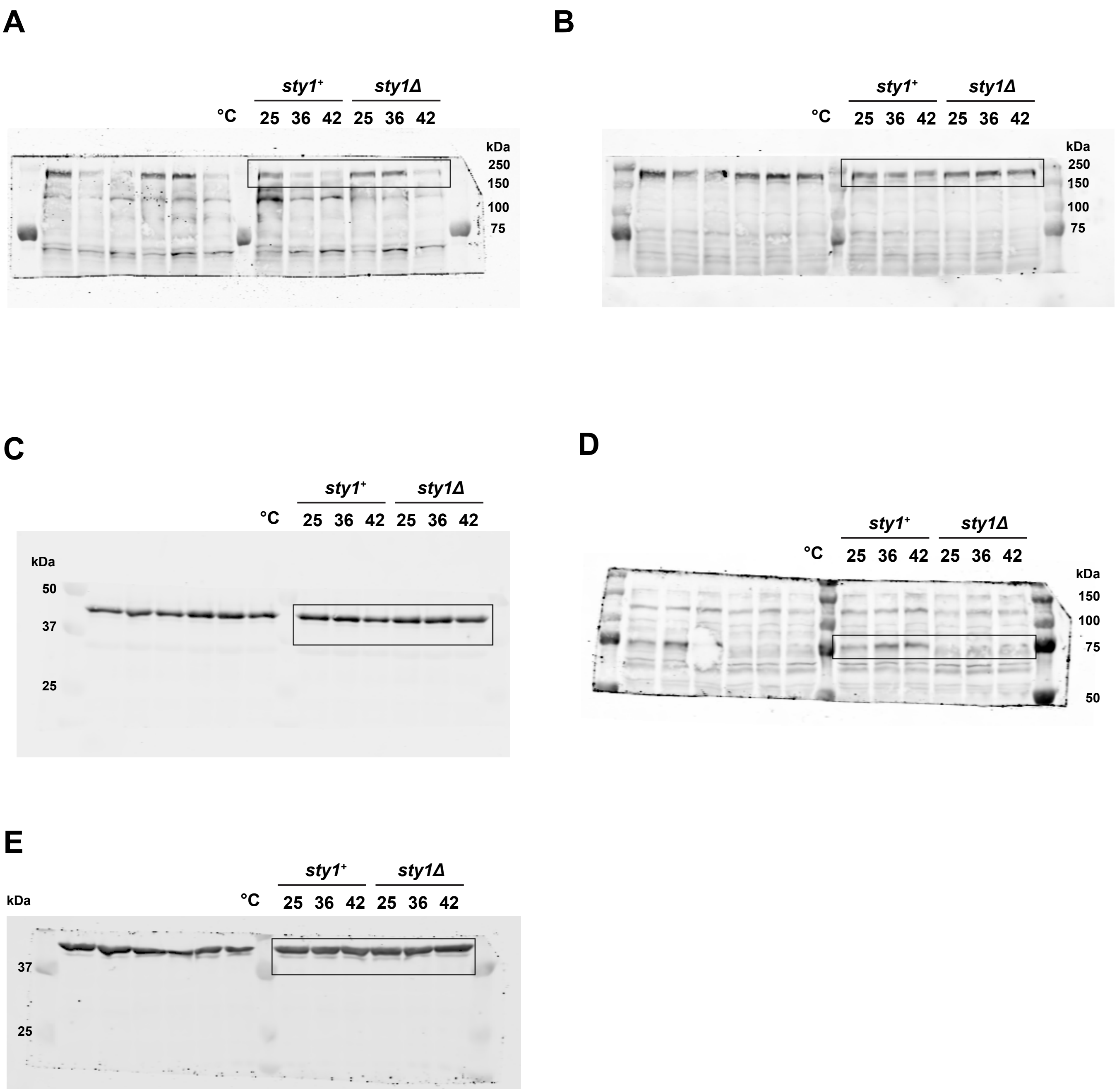

Fig. S7. Uncropped blots from Figure 4G. (A) anti-pGef1-S112. (B) anti-GFP (total Gef1-3YFP). (C) anti-Actin for A-B. (D) anti-Atf1. (E) anti-Actin for D.

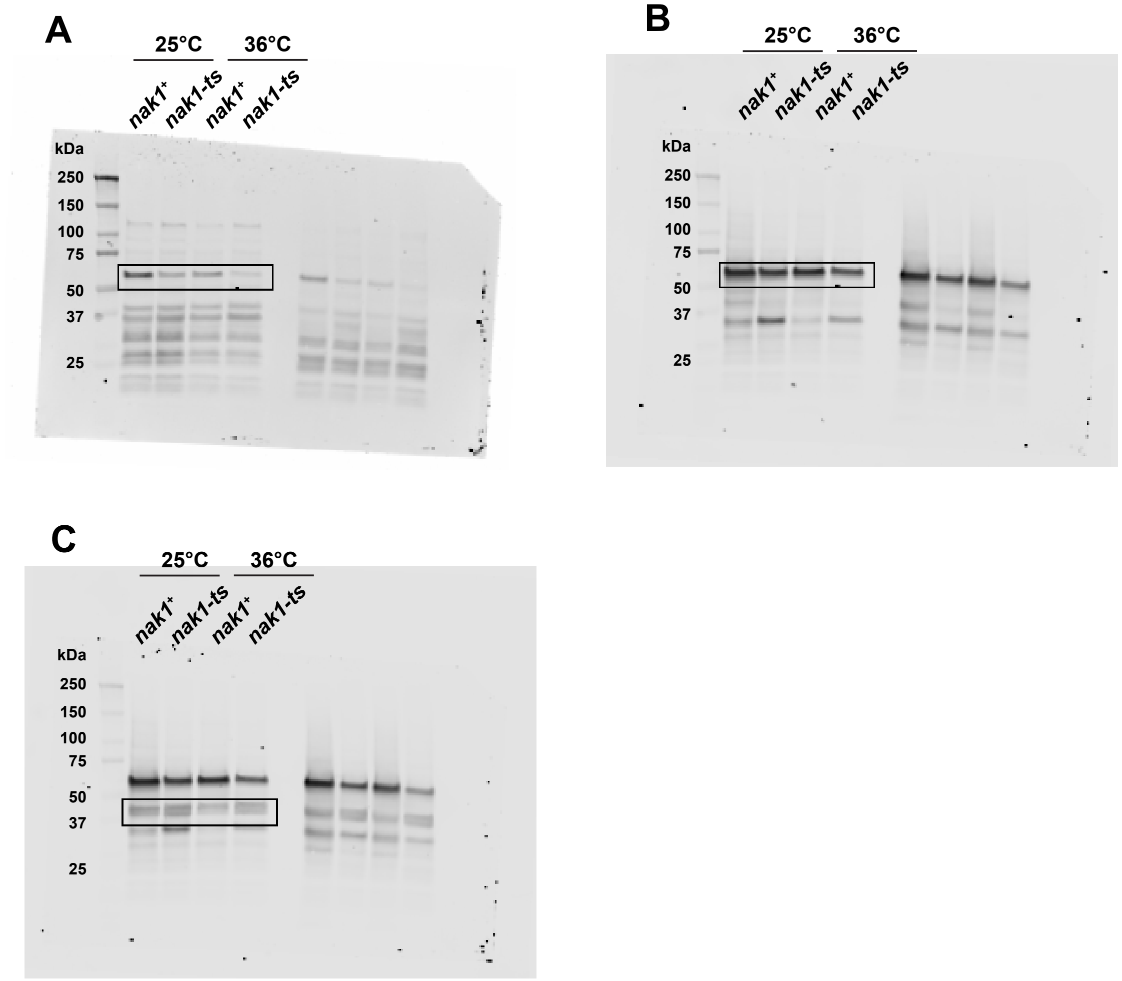

Fig. S8. Uncropped blots from Fig. 5C. (A) antipOrb6-T456. (B) anti-HA (total HA-Orb6as2). (C) anti-Actin for A-B.

Journal of Cell Science • Supplementary information

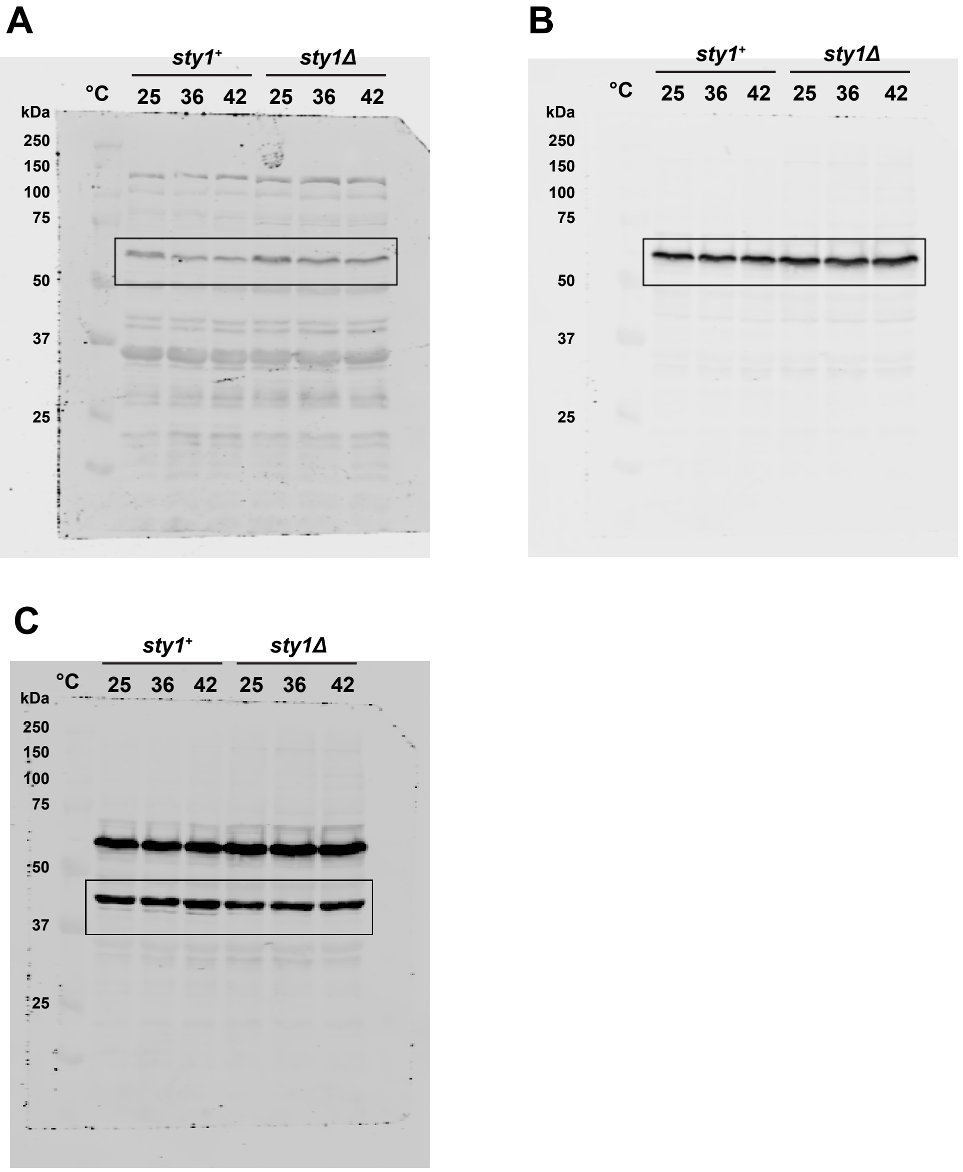

Fig. S9. Uncropped blots from Fig. 6A. (A) anti-pOrb6-T456. (B) anti-HA (total HA-Orb6as2). (C) anti-Actin for A-B.

A

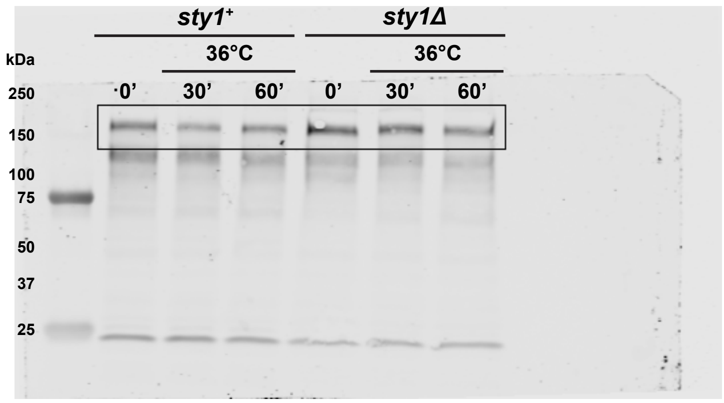

B

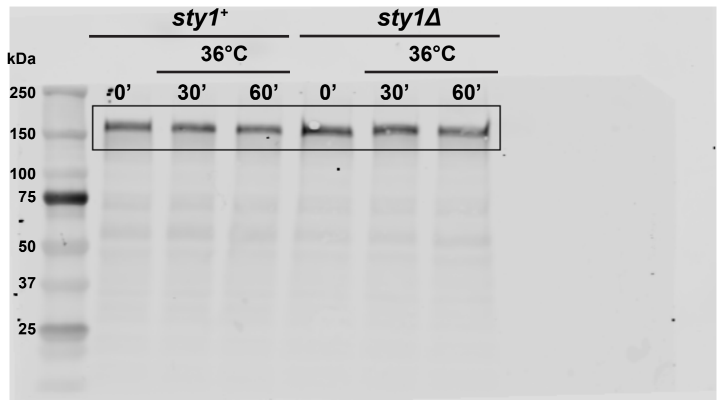

C

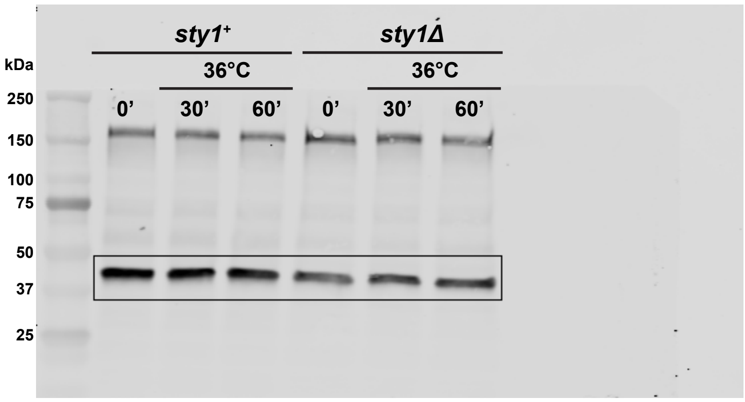

Fig. S10. Uncropped blots from Fig. S5A. (A) anti-pGef1-S112. (B) anti-GFP (total Gef1-3YFP). (C) anti-Actin.

Journal of Cell Science • Supplementary information

Table S1. *S. pombe* strains used in this study.

Strain	Genotype	Source
FV1684	*pabp1-DsRed::KanMX6*	(Nilsson and Sunnerhagen, 2011)
FV2518	*sts5Δ::NatMX6 sts5-3YFP::KanMX6*	(Chen *et al.*, 2019)
FV3194	*sts5Δ::NatMX6 sts5-3YFP::KanMX6 pabp1-DsRed::KanMX6*	This study
FV2522	*sts5Δ::NatMX6 sts5-S86A-3YFP::KanMX6*	(Chen *et al.*, 2019)
FV2527	*orb6Δ::ura4+ pJK148orb6-as2::leu1+*	(Chen *et al.*, 2019)
FV2530	*Wild Type, PN972*	(Leupold, 1949)
FV2644	*Wild Type, PN975*	(Leupold, 1949)
FV2674	*sts5Δ::KanMX6*	(Chen *et al.*, 2019)
FV3192	*sts5Δ::NatMX6 pabp1-DsRed::KanMX6*	This Study.
FV3527	*CRIB-GFP::ura+ ade+ leu+*	(Chen *et al*, 2019), this study
FV3712	*CRIB-GFP::ura+ sty1Δ::kanMX6 ade+ leu+*	This study
FV2206	*gef1Δ::natMX6 gef1-3YFP::kanMX6 ade+ leu+ ura+*	(Chen *et al*, 2019)
FV3634	*gef1Δ::ura+ gef1-3YFP::kanMX6 sty1Δ::kanMX6 ade+ leu+*	This study
FV2444	*sts5Δ::natMX6 sts5-3YFP::kanMX ade6- leu1-32 ura3-D18*	This study
FV3797	*sty1Δ::kanMX6 sts5Δ::natMX6 sts5-3YFP::kanMX ade6- leu1-32 ura3-D18*	This study
FV3343	*nak1-ts (orb3-167)::ura+ orb6Δ::ura+ pJK148HA-orb6-as2::leu+ ade+*	This study
FV3602	*orb6Δ::ura+ pJK148HA-orb6-as2::leu+ ade+*	This study
FV3642	*sty1Δ::kanMX6 orb6Δ::ura+ pJK148HA-orb6-as2::leu+ ade+*	This study
FV3518	*nak1-ts (orb3-167)::ura+ sts5Δ::natMX6 sts5-3YFP::kanMX pabp1-dsRed::KanMX6*	This study
FV2972	*pRep6x sts5Δ::natMX6 sts5-3YFP::kanMX ade-*	(Chen *et al*, 2019)
FV2974	*pRep6x-orb6-T456D sts5Δ::natMX6 sts5-3YFP::kanMX ade-*	(Chen *et al*, 2019)
FV2645	*sts5Δ::natMX6 sts5-HA::kanMX6*	(Chen *et al*, 2019)
FV2649	*sts5Δ::natMX6 sts5-S86A-HA::kanMX6*	(Chen *et al*, 2019)
FV2267	*sts5-3xGFP::NatMX6 dcp1-mCherry::hph*	(Nuñez *et al.*, 2016)
FV2361	*sts5-3xGFP::NatMX6 pabp1-DsRed::KanMX6*	This Study.

Journal of Cell Science • Supplementary information
